# Slab Geometry and Upper Mantle Flow Patterns in the Central Mediterranean From 3D Anisotropic *P*‐Wave Tomography

**DOI:** 10.1029/2021JB023488

**Published:** 2022-05-08

**Authors:** F. Rappisi, B. P. VanderBeek, M. Faccenda, A. Morelli, I. Molinari

**Affiliations:** ^1^ Dipartimento di Geoscienze Università di Padova Padova Italy; ^2^ Istituto Nazionale di Geofisica e Vulcanologia Bologna Italy

**Keywords:** seismic anisotropy, *P*‐wave tomography, Central Mediterranean, subduction zone, mantle dynamics, slab geometry

## Abstract

We present the first three‐dimensional (3D) anisotropic teleseismic *P*‐wave tomography model of the upper mantle covering the entire Central Mediterranean. Compared to isotropic tomography, it is found that including the magnitude, azimuth, and, importantly, dip of seismic anisotropy in our inversions simplifies isotropic heterogeneity by reducing the magnitude of slow anomalies while yielding anisotropy patterns that are consistent with regional tectonics. The isotropic component of our preferred tomography model is dominated by numerous fast anomalies associated with retreating, stagnant, and detached slab segments. In contrast, relatively slower mantle structure is related to slab windows and the opening of back‐arc basins. To better understand the complexities in slab geometry and their relationship to surface geological phenomenon, we present a 3D reconstruction of the main Central Mediterranean slabs down to 700 km based on our anisotropic model. *P*‐wave seismic anisotropy is widespread in the Central Mediterranean upper mantle and is strongest at 200–300 km depth. The anisotropy patterns are interpreted as the result of asthenospheric material flowing primarily horizontally around the main slabs in response to pressure exerted by their mid‐to‐late Cenezoic horizontal motion, while sub‐vertical anisotropy possibly reflects asthenospheric entrainment by descending lithosphere. Our results highlight the importance of anisotropic *P*‐wave imaging for better constraining regional upper mantle geodynamics.

## Key Points

- *P*‐wave tomography resolves three‐dimensional (3D) isotropic and anisotropic structures in the Central Mediterranean
- A 3D reconstruction of the main slabs reveals their complex geometry resulting from pronounced tectonic deformation since the Oligocene
- Azimuth and dip of seismic anisotropy provide new constrains on mantle flow dynamics below the Central Mediterranean

## 1 Introduction

The Central Mediterranean region is an active plate margin characterized by the presence of both oceanic and continental lithosphere. The recent tectonic history is marked by intense seismic and volcanic activity triggered by episodes of continental collision and slab rollback leading to the formation of mountain ranges and extensional basins (Faccenna et al., 2014). Our understanding of the structural heterogeneity and tectonic complexity of this region requires accurate imaging of the subsurface. For this reason, since the late 1990s numerous seismological studies have been carried out to constrain upper mantle structure beneath the Mediterranean region (e.g., Piromallo & Morelli, 2003; Scarfì et al., 2018; Spakman, 1990; Spakman et al., 1993; Spakman & Wortel, 2004). However, despite a few notable exceptions (e.g., Eberhart‐Phillips & Henderson, 2004; Hua et al., 2017; Wei et al., 2019), *P*‐wave tomographic models at regional and global scales generally neglected the phenomenon of seismic anisotropy, approximating the medium as elastically isotropic. Although this assumption simplifies the imaging approach, unmodelled anisotropic heterogeneities generate artifacts that could bias our understanding of the Earth's internal structure and dynamics (Bezada et al., 2016; VanderBeek & Faccenda, 2021). Delays from anisotropic heterogeneities can in fact be as strong, if not stronger, than those from isotropic structure and consequently anisotropy could be mapped to a perturbation in the isotropic velocity (Blackman & Kendall, 1997; Blackman et al., 1996; Grésillaud & Cara, 1996; Kendall, 1994; Lloyd & Van Der Lee, 2008; Sieminski et al., 2007; Sobolev et al., 1999) resulting in misguided interpretations.

Seismic anisotropy in the Central Mediterranean upper mantle has been mostly measured by exploiting surface waves and shear wave splitting. The former generally suffer from poor lateral resolution owing to the long periods used for mantle imaging. The splitting of shear body waves, most commonly SK(K)S, has instead a poor vertical resolution due to their near vertical ray paths. To date, for the Central Mediterranean area, only a single *P*‐wave azimuthal and radial anisotropic tomography study performed in the Alpine region exists (Hua et al., 2017). In this study, teleseismic *P*‐wave delay times are used to infer the isotropic and anisotropic (fabric strength, azimuth, and dip) velocity structures of the Earth's mantle in the Central Mediterranean region. Importantly, our work is the first to consider dipping fabrics in addition to the azimuth and strength of anisotropy which VanderBeek and Faccenda (2021) demonstrated is key to reducing isotropic imaging artifacts. Specifically, our work focuses on the upper mantle where the main source of seismic anisotropy is related to the presence of intrinsically anisotropic minerals, predominantly olivine and to a lesser extent pyroxene. From our anisotropic tomography model, we attempt to answer some fundamental questions regarding Central Mediterranean mantle structure. To what extent could isotropic anomalies be artifacts related to neglected anisotropic heterogeneity? What is the present day configuration of subducting slabs? What is the geometry of mantle flow in relation to the slabs?

### 1.1 Recent Tectonic History

The recent tectonic evolution of the Mediterranean region is characterized by the coexistence of episodes of subduction and collision that expanded and compressed the continental and oceanic lithosphere (Faccenna et al., 2014; Romagny et al., 2020; van Hinsbergen et al., 2014). Bordered at the north by the presence of the Alpine mountain range, whose orogeny dates back to the Late‐Cretaceous (Rosenbaum et al., 2002b), the area is still tectonically evolving. The collision between the African and the Eurasian plates, with the consequent closure of the Tethys Ocean, represents only the beginning of this articulated and complex geological history.

Since the Oligocene, two oceanic trenches surrounding the Alpine‐Dinaric collision have dominated the evolution of the Mediterranean region. To the west the Liguro‐Provençal or Tyrrhenian trench and to the east the Hellenic one. The slow southward retreat of the two trenches begun in the Middle Eocene‐Early Oligocene when the subduction rate of the Ionian slab segments exceeded the convergence rate of the Africa and Eurasia plates. This resulted in a transition from compressional to extensional deformation regime related to the roll back of the oceanic slabs that led to the present day surface and deep structures observed South of the Alps.

From ∼32–30 million years (My) to ∼16–15 My, the south‐eastward migration of the western portion of the Ionian plate triggered the separation of the Corsica‐Sardinia block from the rest of the European continent with a consequent anticlockwise rotation of about 40°. This rotation caused the opening of the Liguro‐Provençal basin (Carminati et al., 2012; Dewey et al., 1989; Faccenna et al., 2004, 2007, 2014; Gueguen et al., 1998; Jolivet et al., 2009; Malinverno & Ryan, 1986; Rosenbaum et al., 2002a; Wortel & Spakman, 2000) and the collision of the Corsica‐Sardinia block with the westernmost part of Adria which gave rise to the Apennines orogeny (Patacca et al., 1993). The retreat of the western portion of the oceanic Ionian plate continues to this day, and from about 15 million years ago (Ma) it has contributed to the fast opening of the Tyrrhenian basin and the southward migration and over‐thrusting of European allochthonous terranes over NE Sicily and North Africa (i.e., Peloritani and Kabylides). More to the West, the Ionian plate experienced a clockwise rotation and westward retreat forming the Alboran‐Betic arc.

Retreat on the eastern side of the Ionian ocean began at 45 Ma and accelerated at 15 Ma as a consequence of the Hellenic slab tearing as documented by mantle tomography (Brun et al., 2016).

**Figure 1:**
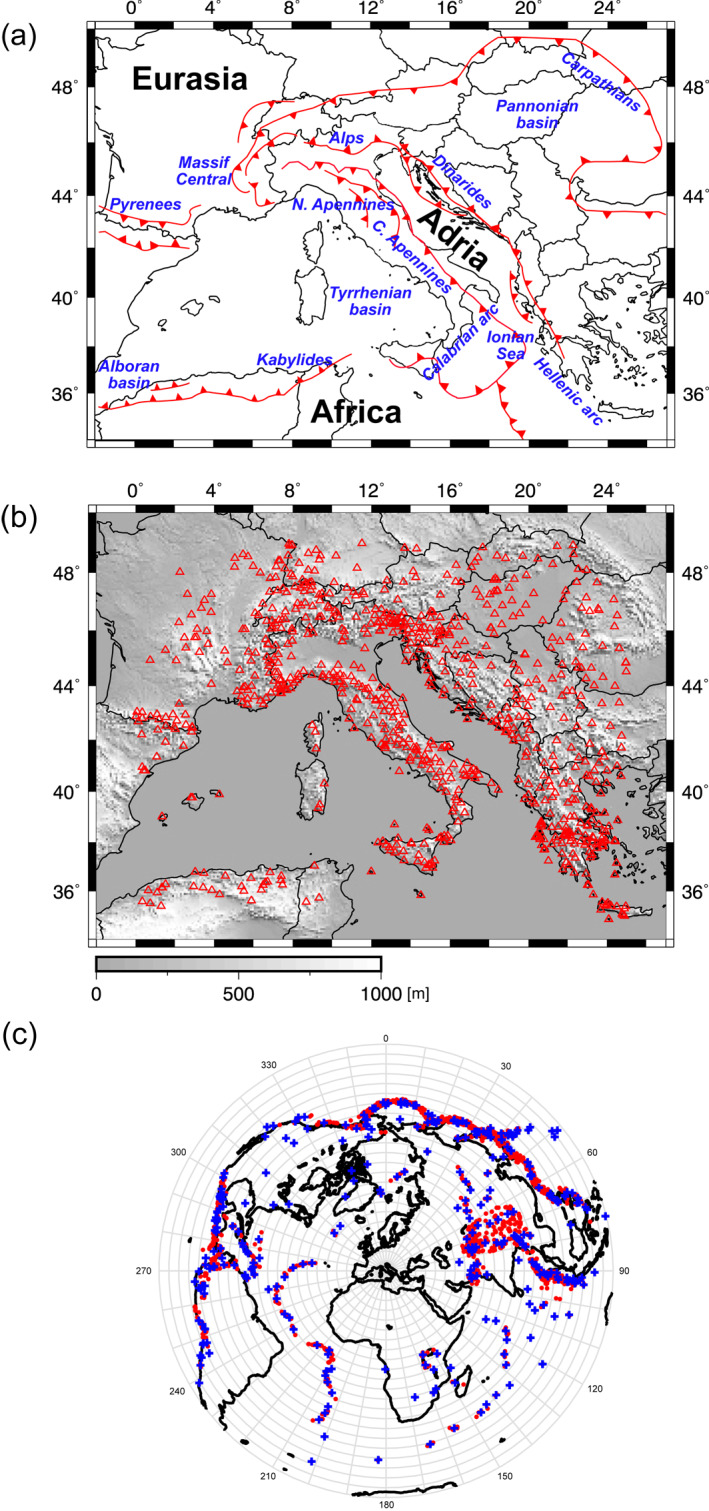
(a) Current plate tectonic setting of the Central Mediterranean adapted from Faccenna et al. (2014). (b) Distribution of seismic stations (red triangles) used in this study. (c) Distribution of teleseismic events considered in this study. All events are shown as red points and binned events used for imaging are depicted with blue crosses. The bins are shown by gray lines and defined by 10° back‐azimuthal and 5° range intervals. Not illustrated are the 50 km depth interval bins.

Faccenna et al. (2014) in their Figure 9 show the evolution of the Mediterranean region starting from 35 Ma. Here, Figure 1a shows the current position of the main trenches in the Central Mediterranean area in relation with the three main tectonic plates Africa, Eurasia, and Adria.

### 1.2 Mantle Structure From Isotropic Tomographic Images

Several seismic tomographic models have been developed in recent decades with the aim of mapping the *P* and *S* wave velocity structures of the mantle in the Mediterranean (Bijwaard et al., 1998; El‐Sharkawy et al., 2020; Hua et al., 2017; Kästle et al., 2019; Koulakov et al., 2009; Lippitsch et al., 2003; Lyu et al., 2017; Piromallo & Morelli, 2003; Spakman et al., 1993; Zhao et al., 2016; Zhu et al., 2012). The various studies indicate that the area is characterized by significant structural heterogeneity. Several fast anomalies interpreted as slab fragments are found with variable lateral and vertical dimensions including continuous slabs reaching the transition zone, hanging slabs and completely detached slabs. While the large‐scale distribution of fast slab anomalies generally agree between studies, the vertical and lateral continuity of these features may vary significantly owing to different methodologies and data sets employed.

Over the years particular attention has been paid to the Alpine area but its complexity and proximity to the Apennines makes the interpretation of the tomographic results particularly difficult. In the Western Alps, for example, some tomographic models have imaged a shallow hanging slab (80–150 km; Kästle et al., 2019) while others have observed a much deeper slab (250–300 km; Hua et al., 2017; Koulakov et al., 2009; Lyu et al., 2017; Zhao et al., 2016). Overall, the Alpine slab appears to be segmented in between Central and Eastern Alps, and dipping at very high angles, especially below the Eastern Alps. The latter feature is particularly problematic as it poses an ambiguity on whether the vertical slab belongs to the European or Adria plates.

The presence of a high‐speed anomaly lying in the Northern Apennines area has been identified by numerous studies (Bijwaard et al., 1998; Giacomuzzi et al., 2011; Kästle et al., 2018; Koulakov et al., 2009; Lucente et al., 1999; Piromallo & Morelli, 1997, 2003; Spakman & Wortel, 2004; Van der Meer et al., 2018; Zhao et al., 2016) but its vertical continuity is still debated. For example, Piromallo and Morelli (2003) show evidence of a continuous slab below Tuscany while Spakman and Wortel (2004) propose that a short (300–400 km) slab is hanging below the Northern Apennines. In the Central Appennines, a slab window of variable vertical extent is imaged ranging from 140 km (Giacomuzzi et al., 2011) to 250 km (Spakman & Wortel, 2004) depth. However, at greater depths the fast Apennine anomaly appears continuous connecting with the Calabrian slab to the south. The high‐velocity Calabrian slab anomaly is observed extending to the transition zone and progressively narrowing at shallower depths as a result of the lateral migration of slab tears (El‐Sharkawy et al., 2020; Giacomuzzi et al., 2012; Neri et al., 2009; Scarfì et al., 2018). Continuing westward, other lithospheric remnants now form the Kabylides slab which extends along the North Africa margin, and the Alboran slab in the Gibraltar area which falls at the far western edge of the study region.

In the Dinaric Alps area several seismic tomographic images (Bijwaard & Spakman, 2000; El‐Sharkawy et al., 2020; Koulakov et al., 2009; Piromallo & Morelli, 2003) found a long slab extending from north to south below the entire mountain range reaching varying depths. For example, El‐Sharkawy et al. (2020) describes a model with a shorter slab in the northern portion (∼150 km) and deeper one (∼300 km) in the southern portion dipping in a northeast direction. Toward the South East, a broad fast anomaly is imaged below the Hellenic arc down to a depth of at least 1,000 km (Piromallo & Morelli, 2003; Zhu et al., 2012).

The most relevant slow anomalies detected in these studies are those present in the shallow mantle beneath areas that over the Cenozoic experienced extensional deformation (e.g., Liguro‐Provencal, Thyrrenian, Aegean, Pannonian basins) or volcanic activity (e.g., Massif Central and Central‐Western Italy; Granet, Wilson, & Achauer, 1995; Granet, Stoll, et al., 1995; Peccerillo, 2017). Other prominent slow velocity anomalies which have no clear connection to surface processes and structures are those imaged beneath the Adriatic and Ionian Seas (Piromallo & Morelli, 2003; Spakman & Wortel, 2004), or below the slab subducting beneath the Western Alps (Zhao et al., 2016).

## 2 Seismic Data

Within our study area, seismic stations are distributed fairly evenly throughout Europe with coverage extending as far south as Northern Africa. However, the marine areas of the Tyrrhenian, Adriatic, and Ionian Seas and the Strait of Sicily remain poorly sampled resulting in uneven seismic data coverage of the Central Mediterranean (Figure 1b).

The data set used for this work consists of direct *P*‐wave delay times reported by the International Seismological Centre EHB bulletin (ISC‐EHB; http://www.isc.ac.uk/isc-ehb/) for the time interval 2000–2018. Delays are measured with respect to AK135 (Kennett et al., 1995) predictions and, as it is common in regional teleseismic tomography (e.g., Lévêque & Masson, 1999; Masson & Romanowicz, 2017), delays are demeaned by event to remove signal originating from structure outside the array. In an effort to select high‐quality data, only International Seismological Centre (ISC) bulletin arrivals that met the following criteria were selected: (a) events with magnitude ≥6; (b) epicentral distance of at least 30° from the edges of the study area; (c) to ensure an event is well recorded across the study area, it was required that at least 90 stations (∼10% of the array) recorded each event; (d) to exclude potentially poor quality instruments, each station must record no less than 10 events; (e) delays with respect to AK135 (Kennett et al., 1995) exceeding four standard deviations (4 s) of the entire delay time data set are considered outliers and excluded (these data compose only 0.34% of all delays). Following preliminary isotropic inversions, the data set was further culled by removing arrivals associated with the tails of the residual histogram that extended beyond three standard deviations of the collective residuals (∼1.8 s). This amounted to a loss of 2% of data (∼1,636 arrivals excluded). Lastly, to reduce biases associated with preferential sampling of certain back azimuths (BAZ), the original 2,918 events were binned in 5° arc distance, 10° BAZ and 50 km depth intervals (Figure 1c). For a given station, delays from events located in the same bin were averaged into a single observation. Our final data set consists of 346 events, 810 stations and includes 89,456 delay times. Mean station delays prior to inversion are shown in Figure S1 in Supporting Information S1. Inversions using raw (i.e., not binned) data with and without the aforementioned outlier arrivals were explored but no significant influence was observed on the tomographic solutions. The main benefit of binning the data was to reduce computational time and minimize the influence of potentially erroneous delays. The final delay time data set has an RMS of 800 ms.

## 3 Seismic Imaging Method

### 3.1 Tomography Algorithm

We use a novel anisotropic seismic imaging method described in detail by VanderBeek and Faccenda (2021) and briefly summarized below. The tomographic algorithm solves simultaneously for perturbations to *P*‐wave slowness (i.e., the inverse of velocity) and three additional parameters that define the anisotropic magnitude, azimuth, and dip in a simplified hexagonally symmetric medium. The method differs from other recent anisotropic *P*‐wave inversion algorithms that include arbitrarily oriented fabrics (e.g., Munzarová et al., 2018) in that our parameterization does not require an anisotropic starting model which could potentially bias results if not sufficiently close to the true solution. Another notable feature of our tomographic method is the use of 3D ray tracing through a user‐defined 3D velocity model that explicitly incorporates elevation (Toomey et al., 1994). As demonstrated by Bodmer et al. (2020), elevation and complex shallow 3D structure can account for more than 1 s of teleseismic delay. Moreover, simply using undamped station static corrections to account for such structure was found to introduce artifacts in the tomographic image. Thus, elevation and shallow velocity variations are best treated explicitly in teleseismic imaging. Lastly, we note that our tomography algorithm can include approximate finite frequency kernels (Schmandt & Humphreys, 2010; VanderBeek & Faccenda, 2021). However, without details regarding the methods and frequency bands used in identifying arrival times in the International Seismological Centre (ISC) catalog, we use the infinite frequency approximation in the present study.

Ray theoretical travel‐times through the modeling volume are computed using a shortest‐path algorithm (Moser, 1991) while the tau‐p method (Crotwell et al., 1999) is used outside the study area where a 1D radial Earth velocity model is assumed. Travel‐times predictions from AK135 are then subtracted from the shortest path time and the resulting residuals for each event are demeaned to yield relative delay time consistent with the observed data. Partial derivatives of the delay times with respect to the model parameters are computed along the discretized ray paths. The resulting system of equations is solved using the LSQR method (Paige & Saunders, 1982) subject to smoothing and damping constraints that are required to regularize the otherwise ill‐posed inverse problem. As a consequence of the evolving 3D ray paths and nonlinear relationship between the travel‐times and anisotropic parameters, multiple iterations are required for the solution to converge.

### 3.2 Starting Model, Discretization and Regularization

For the forward calculation of ray paths and travel‐times, a regular grid with uniform 10 km node spacing was employed. The model domain extends 1,200 km to the east and west of 12.5°E, 1,000 km north and south of 42°N, and 700 km in depth. The initial model contains 3D crustal thickness and isotropic velocity variations from the 1°‐resolution EPcrust (Molinari & Morelli, 2011), which at present is the only crustal model covering the entire area, in addition to elevation from Ryan et al. (2009). The crustal and Moho interfaces in the EPcrust model are linearly interpolated to our model grid and the laterally variable layer velocities in the EPcrust model are interpolated to nodes contained within each layer. Nodes that fall beneath the EPcrust Moho are assigned isotropic mantle velocities from AK135 (Kennett et al., 1995) corresponding to their depth beneath the crust‐mantle interface. Elevation is included by vertically shearing the model grid with additional travel‐time corrections made for differences between the true station elevation and the elevation on the ray tracing grid surface (see Toomey et al., 1994). An Earth flattening transform (Müller, 1971) is applied to the model velocities to account for Earth's curvature in our Cartesian model domain.

We solve for perturbations to mean *P*‐wave slowness and three anisotropic parameters (see VanderBeek & Faccenda, 2021) on a coarser grid with dimensions of 61 × 51 × 20 with uniform 40 km node spacing. Note that purely azimuthally anisotropic inversions were not considered as VanderBeek and Faccenda (2021) demonstrated that these can introduce significant artifacts in areas where dipping anisotropy is to be expected. The resulting perturbations are linearly mapped to the finer model used for travel‐time computations upon each iteration. To further limit the number of inversion parameters, anisotropic perturbations were restricted to the upper 400 km where mineral physics predicts mantle anisotropy to be most significant (Karato et al., 2008) and where there is the best ray crossing. Furthermore, inversions without depth‐restricted anisotropy did not significantly improve the fit to the data nor appreciably alter the final image. Our isotropic inversions converge after three iterations (where one iteration comprises the forward computation of ray paths and delay times and subsequent inversion for new model perturbations) while the anisotropic inversions converge after six iterations.

The selection of regularization parameters that enforce the Laplacian spatial smoothness of the model perturbations (i.e., smoothing factor, *λ*_*s*_) and limit the norm of the model perturbational vector (i.e., damping factor, *λ*_*d*_) is the most subjective aspect of a tomographic inversion. To avoid preferentially making isotropic perturbations whose partial derivatives are generally larger in magnitude relative to the anisotropic variables, the slowness regularization equations are inversely weighted by the starting model slowness. In this way, damping and smoothing is applied to fractional changes in model parameters which are expected to be on the order of 1% for both isotropic and anisotropic perturbations. To identify appropriate regularization values, we constructed L‐curves (e.g., Aster et al., 2018) which plot the squared‐norm of the data residual vector against the squared‐norm of the model perturbational vector. Ideal solutions are considered those near the corner of the L‐curve where an increase in model norm does not result in an appreciable decrease in data residuals. Further discussion about the choice of the regularization parameters is addressed in Text S1 in Supporting Information S1. The L‐curves are shown in Figure S2 in Supporting Information S1. The parameters adopted for our preferred isotropic and anisotropic solutions are listed in Table S1 in Supporting Information S1.

Event and damped station correction terms are also included in the inversion. Event statics account for hypocentral errors and structure sampled outside the imaging volume. Station corrections are traditionally used to account for shallow structure that cannot be resolved by the teleseismic data (e.g., elevation changes and crustal heterogeneity). Considering our starting model contains elevation and 3D crustal velocities, we follow Bodmer et al. (2020) and solve for damped station correction terms. The damping factor is chosen such that the RMS station correction is ∼300 ms. This corresponds to a ∼3.75% change in average crustal velocity or 8 km change in crustal thickness.

The RMS delay time from our preferred isotropic solution (referred to as iso‐NEWTON21 in reference to the name of the European Research Council grant funding this work; see Data Set S1 in Supporting Information S1) is 500 ms corresponding to a 61% variance reduction in the initial RMS delay time. In comparison, our preferred anisotropic solution, ani‐NEWTON21 (Data Set S2 in Supporting Information S1), has an RMS delay time of 488 ms corresponding to a 63% variance reduction. The similar data fit offered by the two models may reflect the true error in our delay time data set and more accurate delay times may allow us to better distinguish between them in the future. While the small improvement in data fit alone does not justify the inclusion of additional anisotropic parameters, we assert that the ani‐NEWTON21 model is the more optimal solution for the following reasons. (a) While the anisotropic model has more free parameters, it is the simpler solution. The total norm of the fractional velocity perturbations (i.e., change in isotropic velocity normalized by the starting model value) and anisotropic magnitude perturbations can be used as an indication of model complexity with higher values corresponding to a greater magnitude and/or number of anomalies. The norms are comparable as both fractional velocity anomalies and anisotropic magnitude describe perturbations to seismic propagation velocity and are expected to be similarly valued (i.e., a few percent). From the L‐curves (Figure S2 in Supporting Information S1), it is clear that anisotropic models consistently fit the data better with less heterogeneity. (b) Unlike the isotropic model, the anisotropic solution can explain many patterns observed in independent SKS splitting parameters and is thus more consistent with observations. (c) As evidenced by numerous SKS splitting studies, the central Mediterranean is underlain by rather complex anisotropic structure and it is known that *P*‐wave travel‐times are particularly sensitive to anisotropy (Sieminski et al., 2007) such that assuming an isotropic Earth can lead to significant imaging artifacts and possible erroneous interpretations (e.g., Bezada et al., 2016; VanderBeek & Faccenda, 2021). Therefore, neglecting anisotropy in body wave imaging is problematic and both isotropic and anisotropic inversions should be conducted to understand the nature of mantle heterogeneity. (d) Both ours and the synthetic tests of VanderBeek and Faccenda (2021) demonstrate both isotropic and anisotropic heterogeneity can be resolved by teleseismic *P*‐weave delays and that there is not a one‐to‐one trade off in these parameters. Furthermore, true isotropic structure is not prone to yielding anisotropic artifacts. However, anisotropic structure can generate significant isotropic artifacts (VanderBeek & Faccenda, 2021). Thus, isotropic features in the anisotropic model are likely to be more robust.

### 3.3 Model Resolution

Anisotropic imaging with teleseismic delay times has some important limitations. We summarize these issues in Text S2 in Supporting Information S1 and present in the next lines metrics for model resolution and results of synthetic tests to evaluate the effects of these limitations on our results.

Directly assessing model resolution for large scale tomographic problems remains a challenge. The large number of free parameters generally prohibits the direct computation of resolution matrices and nonlinear inversion methods capable of systematically exploring model space quickly become computationally infeasible. Hence, three more indirect measures of model fidelity are tested, (a) ray density and directional sampling metrics, (b) synthetic reconstruction tests, and (c) predictive capability of the tomographic model.

The derivative weight sum (DWS; Toomey & Foulger, 1989) is the summation of travel‐time partial derivatives with respect to slowness at each perturbational node. As demonstrated by Zhang and Thurber (2007), the derivative weight sum (DWS) provides an indirect estimate of parameter resolution attaining higher values in more densely sampled regions of the model.

The derivative weight sum (DWS) lacks information regarding how directionally well‐sampled are the model parameters which is important for assessing resolution of anisotropic structure. To assess directional bias, we use the azimuthal mean resultant length (AMRL; Fisher, 1995; Zhang et al., 2009) defined as the length of the vector resulting from an averaging of the x‐ and y‐components of all ray segment unit vectors sampling a given perturbational node. Maps of the DWS (Figure S3 in Supporting Information S1) and AMRL (Figure S4 in Supporting Information S1) for our study are shown in Supporting Information S1 together with their discussion in Text S2 in Supporting Information S1.

Directional sampling in the vertical plane is poor compared to the azimuthal plane owing to the steep incidence angles of teleseismic wavefronts. Figure S5 in Supporting Information S1 shows the mean incidence angles of rays throughout the imaging volume which primarily vary as a function of depth with values ranging between 20° and 40°. Despite the limited sampling of incidence angles, good azimuthal coverage is sufficient to resolve dipping anisotropic fabrics (VanderBeek & Faccenda, 2021) but results in limited vertical resolution of anisotropy as shown by our synthetic inversions discussed below.

A number of synthetic inversions is performed to address potential imaging problems highlighted in Text S2 in Supporting Information S1 and to evaluate isotropic and anisotropic parameter resolution in general. Ray theoretical synthetic delay times are predicted for various test models using the same station‐event pairs defined in our binned data set (Section 2) with event demeaning to be consistent with the true observations. Random errors from a normal distribution with a standard deviation of 450 ms (i.e., a value comparable to the RMS‐error of our preferred tomographic solutions) are added to the synthetic data sets. Unless otherwise noted, all synthetic tests are performed using the preferred anisotropic inversion parameters listed in Table S1 in Supporting Information S1.

**Figure 2:**
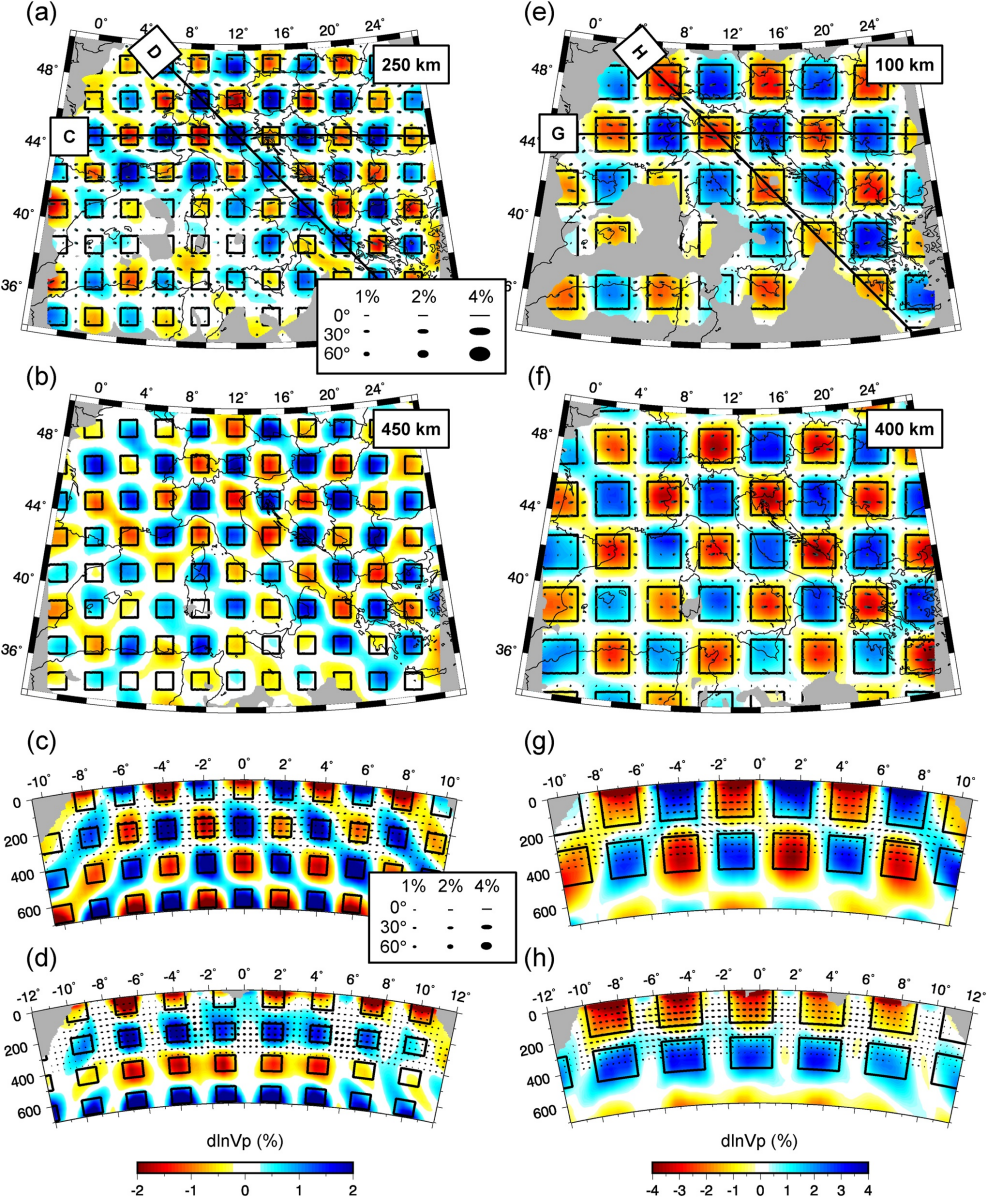
Isotropic checkerboard test results. Reconstruction of 100 km cubic anomalies at (a) 250 km and (b) 450 km depth with cross‐sections shown in (c) and (d) along the corresponding profiles drawn in (a). Reconstruction of 200 km cubic anomalies are shown at (e) 100 km and (f) 400 km depth with cross‐sections shown in (g) and (h) along the corresponding profile lines in (e). In each panel, the location of the true anomalies are outlined in black and defined by alternating isotropic perturbations of ±4%. While no anisotropic structure is present in the target model, the inversion does introduce some anisotropic perturbations. Anisotropy is represented by ellipse symbols where the major axis of the ellipse parallels the fast‐direction and the minor axis scales linearly with the symmetry axis dip into the view plane such that fabrics parallel and normal to the cross‐sections plot as lines and circles, respectively. In (a–b) and (e–f), small black quivers on the ellipses indicate the direction of dip into the earth. Legends in (a) and (c) depict reference ellipses for different fabric strengths and dips in the horizontal and vertical cross‐sections, respectively. Areas of poor data coverage are masked in gray. Note the change in colorscale between panels a–d and e–h. While both synthetic models were defined with the same anomaly amplitudes, the amplitudes of the smaller blocks are more underestimated and a narrower value range is used so that the geometry of the imaged blocks is easily observed.

To assess resolution of isotropic structure and its trade‐off with anisotropic parameters, checkerboard reconstruction tests were performed for purely isotropic cubic anomalies with alternating amplitudes of ±4% and dimensions of 100 km (Figures 2a–2d) and 200 km (Figures 2e–2h). It is found that lateral and vertical variations in isotropic structure on the scale of at least 100 km are well‐resolved in areas where the DWS >∼100. However, as is common in ray‐theoretical tomography, amplitudes are generally under‐recovered. The degree to which the anomaly amplitudes are underestimated depends in part on their spatial extent. We recover ∼50% of the 100 km^3^ block amplitudes and 75%–80% of the 200 km^3^ blocks. Despite the potential of the checkerboard‐pattern isotropic anomalies to impart a directional dependence in delay times, only minor anisotropic anomalies (generally <1%) are imaged indicating minimal leakage of truly isotropic heterogeneity into anisotropic parameters. Lastly, it is worth noting that purely isotropic inversions for these models did not improve amplitude recovery or alter the solution in any appreciable way.

**Figure 3:**
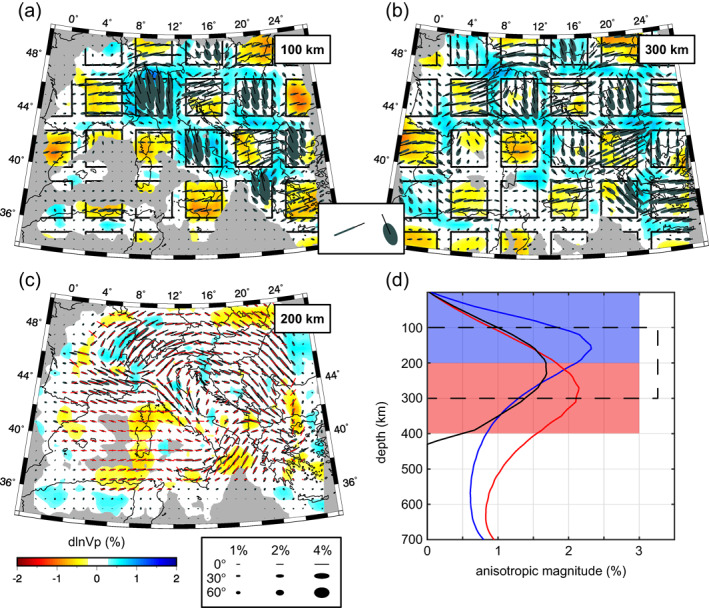
Anisotropic checkerboard tests. Reconstruction of 300 × 300 × 200 km anisotropic blocks centered at (a) 100 km and (b) 300 km depth. Location of true anomalies are outlined in black and contain 6% *P*‐wave anisotropy. The azimuth of anisotropy alternates between 22.5° and −67.5° while the dip varies from 0° to 45°. Symbols corresponding to the true fabrics are shown in the legend between panels (a) and (b). In (c), we plot the recovery of a 200 km‐thick anisotropic layer centered at 200 km depth in which fast‐axes parallel the fast SKS splitting direction and anisotropic magnitude is proportional to the split time (see Section 3.3 for details). True orientations and magnitudes are shown by red quivers. No isotropic structure is present in the test models though the inversions introduce some isotropic artifacts. The average anisotropic magnitude as a function of depth is shown in (d) for the solutions in (a; blue curve) (b; red curve) and (c; black curve). The true depth distribution of anisotropy is shown by the shaded blue and red regions for models (a) and (b) and the dashed black line for model (c). Colorscale and anisotropy symbol legend (see Figure 2 for description) are shown beneath (c).

To investigate to what extent anisotropic heterogeneity can be isolated, additional checkerboard tests were performed for purely anisotropic anomalies. Two models were considered composed of 300 × 300 × 200 km anisotropic domains centered at (a) 100 km (Figure 3a) and (b) 300 km depth (Figure 3b). Each block contains 6% *P*‐wave anisotropy with fast axis azimuths alternating between 22.5° and −67.5°, while the dip varies between 0° and 45°. To visualize the effects of vertical smoothing, anisotropic perturbations were not limited to the (0 km, 400 km) depth interval as in our preferred solution. Strong lateral changes in anisotropic fabric are well‐imaged at the scale of ∼300 km where the AMRL >∼0.5. On average, 50%–70% of the anisotropic amplitudes are recovered. The median error in fast axis azimuths for both tests is 14° and 10° for the dip using angular errors on a (0°, 90°) interval. As seen in Figure 3d, the anisotropic domains are reasonably well‐localized in depth with the inversions recovering the strongest anisotropic magnitudes at depths coinciding with the anomaly centers. However, the peak recovered magnitude for the shallow block is offset deeper by ∼50 km. Vertical smoothing does smear some anisotropic structure throughout the upper mantle. Because of the poor sampling of incidence angles, synthetic tests involving strong vertical changes in anisotropy orientations are poorly resolved and tend to yield a depth‐averaged fabric. This explains why our preferred anisotropic solution presented in Section 4.2 does not vary significantly with depth. Compared to the isotropic checkerboard tests, it is clear that anisotropic parameters are generally less‐well resolved and yield more pervasive isotropic artifacts with magnitudes around 1% (Figures 3a and 3b).

To further investigate the resolution of dipping fabrics, we performed two additional synthetic tests (Figures S6 and S7 in Supporting Information S1) discussed in Text S3 in Supporting Information S1.

Checkerboard tests represent rather complex and geologically unrealistic structure. As discussed by VanderBeek and Faccenda (2021), strong lateral variations in anisotropic fabrics are ideal anomalies for regional teleseismic data as their signals are not easily removed by demeaning. To determine if our data set is capable of constraining more realistic and smoother anisotropic heterogeneity, we construct a synthetic test based on SKS splitting observations from the area (Figure 3c). The target model contains a 200 km thick azimuthally anisotropic layer centered at 200 km depth; parameters do not vary with depth in the layer. The fast‐axis azimuths within the layer are linearly interpolated from the station‐averaged fast SKS polarization directions in the Becker et al. (2012; updated 6 December 2020) database. Station‐averaged SKS split times (DT) are also interpolated to the layer and converted to *P*‐wave anisotropic magnitude by the expression 1.51*v*_*s*_ *DT*/200, where *v*_*s*_ is the mean shear‐wave velocity between 100 and 300 km depth in the AK135 velocity model (4.55 km/s) and the factor of 1.51 is the ratio between *P*‐ and *S*‐wave anisotropy measured for a peridotite sample by Kern (1993). Relative delay times accurately reconstruct such an anisotropic layer (Figure 3c). The median azimuthal error in the solution is 12°. The depth of the anisotropic layer is correctly imaged albeit with vertical smoothing throughout the upper 400 km and ∼50% of the true anisotropy amplitude are recovered (Figure 3d). If SKS splits reflect the complexity of mantle anisotropy beneath the study area, then relative delay time tomography should yield an unbiased recovery of such heterogeneity.

**Figure 4:**
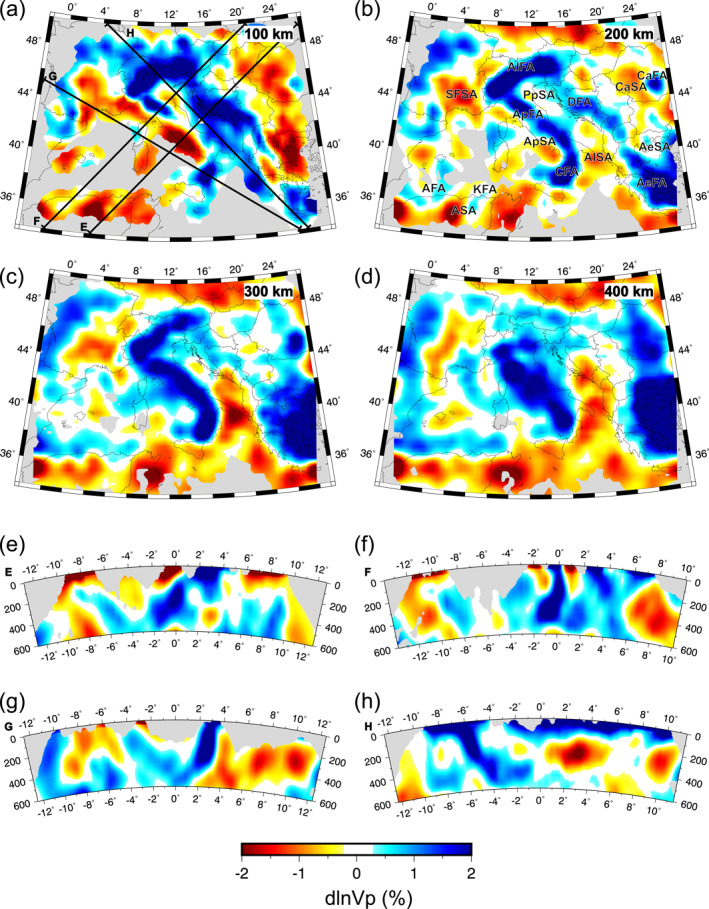
The iso‐NEWTON21 model. Horizontal cross‐sections are shown at (a) 100 km, (b) 200 km, (c) 300 km, and (d) 400 km depth. Vertical cross‐sections are shown in (e–f) along the corresponding profile lines drawn in (a). Major anomalies discussed in the text are labeled in (b). Isotropic anomalies are plotted with respect to starting model. Areas of poor data coverage are masked in gray. See text for description of acronyms.

**Figure 5:**
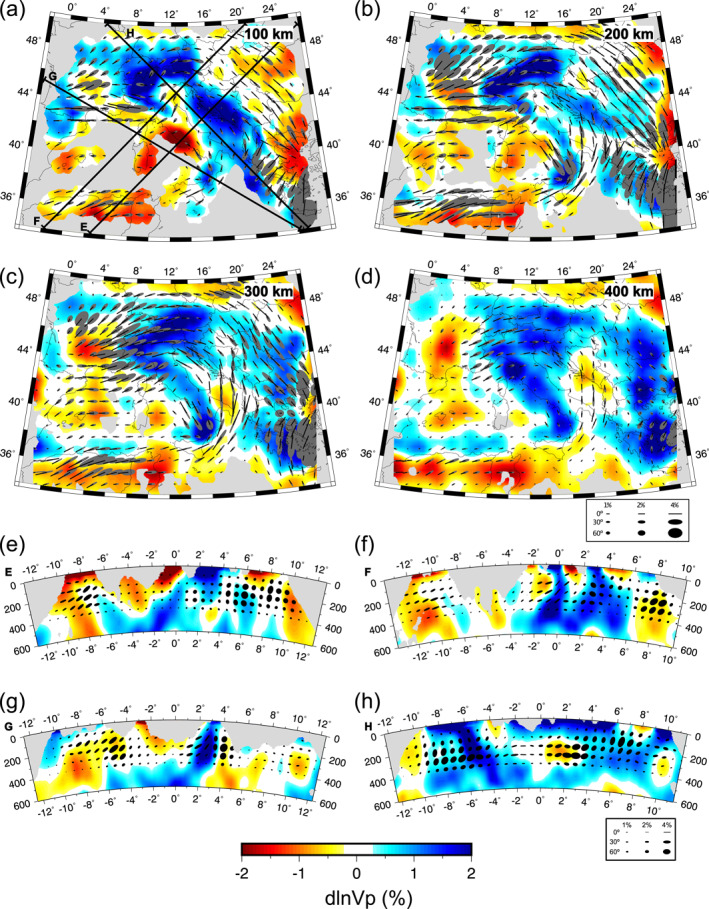
The ani‐NEWTON21 model. Horizontal cross‐sections are shown at (a) 100 km, (b) 200 km, (c) 300 km, and (d) 400 km depth. Vertical cross‐sections are shown in (e–f) along the corresponding profile lines drawn in (a). Isotropic anomalies are plotted with respect to starting model. Anisotropy is plotted using ellipses as described in Figure 2. Areas of poor data coverage are masked in gray.

To further explore possible trade‐offs between isotropic and anisotropic parameters, synthetic inversions were ran aimed at reconstructing our preferred isotropic (Figure 4) and anisotropic (Figure 5) models presented in Section 4. To test if velocity anomalies present in our preferred isotropic model could yield erroneous anisotropy, delays predicted through this solution were inverted for both isotropic and anisotropic parameters. Isotropic anomalies were faithfully recovered with minimal (generally <1%) anisotropic perturbations (Figure S8 in Supporting Information S1). Delays predicted through our preferred anisotropic model were then inverted without (Figure S9 in Supporting Information S1) and with (Figure S10 in Supporting Information S1) anisotropy. Neglecting anisotropy yielded a solution nearly identical to our preferred isotropic model (Figure 4 vs. Figure S9 in Supporting Information S1) indicating that the anisotropic heterogeneity can be mapped into significant isotropic perturbations. The anisotropic inversion (Figure S10 in Supporting Information S1) accurately recovered fabric strength and orientations (median azimuthal and dip error of 8° and 6°, respectively) as well as isotropic velocity structure.

Lastly, the fidelity of our anisotropic model is assessed by its ability to predict independent SKS splitting observations. Numerous SKS splitting studies have been carried out across Europe (see compilations by Becker et al., 2012; Wüstefeld et al., 2009) and the mantle anisotropic structure imaged by *P*‐waves should be consistent with these observations. A detailed description of the method used to model the effect of anisotropy on a SKS waveform is addressed in Text S4 in Supporting Information S1. The results of this analysis are presented in relation to the recovered *P*‐wave anisotropy in Section 5.2, where we show that our *P*‐wave model predicts most of the observed SKS splitting patterns.

## 4 Tomographic Model

We present two tomographic models for the central Mediterranean region; one is purely isotropic (Figure 4) while the other includes 3D anisotropic heterogeneity (Figure 5). We first describe the primary *P*‐wave speed perturbations in our preferred isotropic solution and then consider how the inclusion of anisotropic parameters modifies these anomalies. Lastly, we describe anisotropic patterns beneath the Mediterranean as seen by teleseismic *P*‐wave delays. We focus on mantle structures deeper than ∼100 km and shallower than ∼700 km where we have the best data coverage.

### 4.1 Purely Isotropic Solution

Our preferred isotropic tomographic model, iso‐NEWTON21, is presented in Figure 4 (see Figures S11a and S11b in Supporting Information S1 for additional maps at 500 and 600 km depth and Figures S12a–S12f in Supporting Information S1 for broader colorscale limits). It contains a number of fast anomaly features broadly consistent with previous seismic imaging studies. Specifically, the large‐scale high‐velocity zones underlying the Apennines, Calabrian arc, Alps, and Dinarides.

A prominent fast velocity feature is imaged extending along the Apennines (Apennines Fast Anomaly, ApFA) that at shallow depths (<200 km) is divided into a northern and southern segment by the Apennines Slow Anomaly (ApSA; Figure 4a). At depths greater that ∼150 km, the ApFA is continuous throughout Italy and curves toward Sicily becoming the Calabrian Fast Anomaly (CFA; Figures 4b–4d). Together, the ApFA and Calabrian Fast Anomaly (CFA) form a single hook‐shaped high‐speed belt throughout the Italian peninsula. This high‐speed belt dips toward the Tyrrhenian Basin and can, in most places, be traced to 600 km depth (Figures 4e–4g).

Further north, the Alpine Fast Anomaly (AlFA) extends SW‐NE across the entire Alps. Near 400 km depth (Figure 4d) the AlFA amplitudes become less laterally continuous separating into eastern‐ and western‐AlFA segments. In cross‐section H (Figure 4h), the central AlFA dips steeply to the southeast in the upper 400 km and then becomes horizontal at ∼500 km. This trend is not evident in the western and eastern AlFA where the anomaly appears more vertically oriented.

At shallow depths (<200 km) the elongated Dinaric Fast Anomaly (DFA) can be traced from the eastern Alps to Greece following the trend of the Dinarides. The amplitude of this feature quickly diminishes below ∼300 km (Figures 4b–4d). The DFA generally appears to dip eastward in cross‐section (Figures 4e and 4f).

Notable fast anomalies are imaged also underlying the Aegean Sea and Hellenic Peninsula (AeFA) and Carpathian Mountains (CaFA). Another high‐speed feature is the Kabylides Fast Anomaly (KFA) along the North African margin that follows the Atlas Mountains and appears to connect with the Alboran Fast Anomaly (AFA) present eastward of the Strait of Gibraltar. The AeFA is characterized by high amplitudes (>2%) dipping northeastward and extending beneath Greece. The CaFA manifests as a small circular (∼200 km diameter) anomaly at 100–200 km depths becoming more laterally expansive around 300–400 km depth at which point it appears to connect with the AeFA. The KFA and AFA are weaker anomalies (0.5%–1%) observed at depths greater than 200–300 km and down to ∼600 km (Figures 4e and 4f). Below ∼400 km depth, the KFA appears to connect with the deep portion of the ApFA (Figure S11 in Supporting Information S1). The AeFA, CaFA, KFA, and AFA are notable given their spatial correspondence with prominent geologic features. However, we note that they are near the edges of our study area and may be less‐well resolved.

Several slow anomalies can also be observed in the iso‐NEWTON21 model. Among them is the previously mentioned central‐ApSA, located in central‐west Italy along the Tyrrhenian coast. This anomaly interrupts the lateral continuity of the ApFA creating a well‐known window (e.g., Kästle et al., 2018; Lucente et al., 1999; Piromallo & Morelli, 2003; Van der Meer et al., 2018) that separates the northern and southern ApFA at shallow depths; below ∼200 km the central‐ApSA is no longer observed and the ApFA is continuous (Figures 4a–4d). A prominent slow anomaly is imaged in the south of France (SFSA), in correspondence of the Massif Central already detected by Granet, Wilson, and Achauer (1995) and Granet, Stoll, et al. (1995). The SFSA stretches in the NW‐SE direction in the first 100 km and, although with ever smaller magnitudes and dimensions, it persists down to 400 km of depth. A weaker (<1%) smaller‐scale slow anomaly can be identified at shallow depths (<200 km) beneath the Po Plain (PpSA). A strong slow anomaly is detected at the boundary beneath the Adriatic and Ionian seas (AISA) off the southeastern tip of Italy where velocities are reduced by <−2% at ∼300 km (Figure 4c). Curiously, this feature is not spatially associated to any superficial geologic phenomenon. While the Adriatic and Ionian seas (AISA) is observed in other isotropic tomographic models (e.g., Li et al., 2008; Piromallo & Morelli, 2003; Spakman & Wortel, 2004), its origin is unclear. The Carpathian and Aegean Sea Slow anomalies (CaSA and AeSA, respectively) are two laterally extensive low‐velocity zones restricted to the upper 200 km of our isotropic result. Lastly, we note the African Slow Anomaly (ASA) south of and generally parallel to the KFA. Above 100 km, the ASA manifests as three circular high magnitude anomalies (<−2%) and becomes more laterally continuous with depth.

### 4.2 Anisotropic Solution

Our anisotropic model, ani‐NEWTON21, is presented in Figure 5. Additional maps are shown at 500 and 600 km depth in Figures S11c and S11d in Supporting Information S1, with broader colorscale limits in Figures S12g–S12l in Supporting Information S1 and differences in isotropic anomaly amplitudes between iso‐NEWTON21 and ani‐NEWTON21 in Figure S13 in Supporting Information S1. The first‐order effect of including anisotropic parameters in the inversion is to reduce the magnitude of isotropic anomalies (Figure S13 in Supporting Information S1). This is the result of mapping isotropic structure into the anisotropic parameters. The amplitude reduction is generally more significant for low‐velocity relative to high‐velocity zones. This is mostly due to the anisotropic symmetry system assumed for the mantle. Upper mantle anisotropy is largely the result of the preferential alignment of olivine crystals which tend to generate fabrics characterized by hexagonal symmetry with a single fast and two slower *P*‐wave speed propagation directions (Karato et al., 2008). Because the anisotropic fabrics in our model tend to be oriented sub‐horizontally, steeply propagating teleseismic *P*‐waves preferentially sample slower directions of the velocity surface resulting in an overall slower model requiring smaller reductions in isotropic velocity. This effect is particularly evident for the SFSA and Adriatic and Ionian seas (AISA) whose amplitudes are strongly reduced in coincidence with regions of elevated *P*‐wave anisotropy (∼2%; Figure 5) and strong shear wave splitting (∼1.5 s; Becker et al., 2012). At greater depths (300–400 km), the SFSA appears more spatially concentrated occupying a roughly circular region defined by slightly stronger *P*‐wave speed reductions with respect to iso‐NEWTON21. A similar effect is seen around the Pannonian Basin and the ASA where strong sub‐horizontal anisotropy is present. In contrast, the amplitude of the central‐ApSA in ani‐NEWTON21 is similar to iso‐NEWTON21 as it occupies a region of relatively weak *P*‐wave anisotropy.

The geometry of the high‐velocity features in our ani‐NEWTON21 model remains largely unchanged but we do observe a general reduction in magnitude (Figure S13 in Supporting Information S1). This is most evident in the central‐ApFA and in the AeFA. The amplitude reduction associated with the central‐ApFA is coincident with an area of relatively weak *P*‐wave anisotropy while the amplitude reduction in the Aegean region is associated with the presence of strong NNW‐trending anisotropy dipping moderately (>30°) to the north.

Model ani‐NEWTON21 exhibits a large‐scale circular pattern in anisotropy azimuth around the Italian peninsula most evident in map view at 200 and 300 km depth in Figures 5b and 5c. Other primary anisotropic structures recovered by the inversion include the one located beneath the Alps where the fast axes of *P*‐waves coincide with the elongation direction of the AlFA, trending WSW‐ENE. Near the Eastern Alps the azimuths gradually turn toward SE‐NW. These fabrics persist throughout the Dinarides and Pannonian Basin. The fast axes continue to rotate clockwise becoming more N‐S in Adriatic Sea and SW‐NE around Calabria. Throughout northern Africa fast axes aligned E‐W are observed. On a smaller scale, anisotropic azimuths normal to the strike of the adjacent fast anomalies are imaged immediately eastward of the northern‐ApFA and northward of the Calabrian Fast Anomaly (CFA).

A diversity of dip angles are also observed in our tomographic model. Near‐horizontal fabrics are widespread in the Ionian and Adriatic Seas as well as throughout the Dinarides and Pannonian Basin. In contrast, more steeply dipping fabrics are observed around the western edge of the Alps near the SFSA and in the areas surrounding the Northern Apennines, Aegean Sea and ASA. The steepest dipping fabrics are observed within the CFA and AeFA (>30°).

The magnitude of *P*‐wave anisotropy recovered throughout the study area is generally 2%–3%. Notable areas of weak anisotropy include the AlFA and central‐ApFA/ApSA, while the strongest anisotropy is observed in association with the circular pattern around the southern edge of Italy and the AeFA.

## 5 Discussion

We focus our discussion on the geodynamic interpretation of the anisotropic model and how it differs from the purely isotropic solution and previously published isotropic and anisotropic models. First, we discuss the isotropic anomalies recovered by the ani‐NEWTON21 model by presenting a 3D reconstruction of lithospheric slabs geometry, and by providing interpretations for the low‐velocity features (Section 5.1). Following a comparison between the anisotropic component of our *P*‐wave model to anisotropy patterns derived from other studies (Section 5.2), we take advantage of recently published geodynamic models to discuss the nature of upper mantle flow in the Central Mediterranean in light of the new anisotropic patterns (Section 5.3).

### 5.1 A 3D Model of Central Mediterranean Slabs and Origin of Low‐Velocity Zones

**Figure 6:**
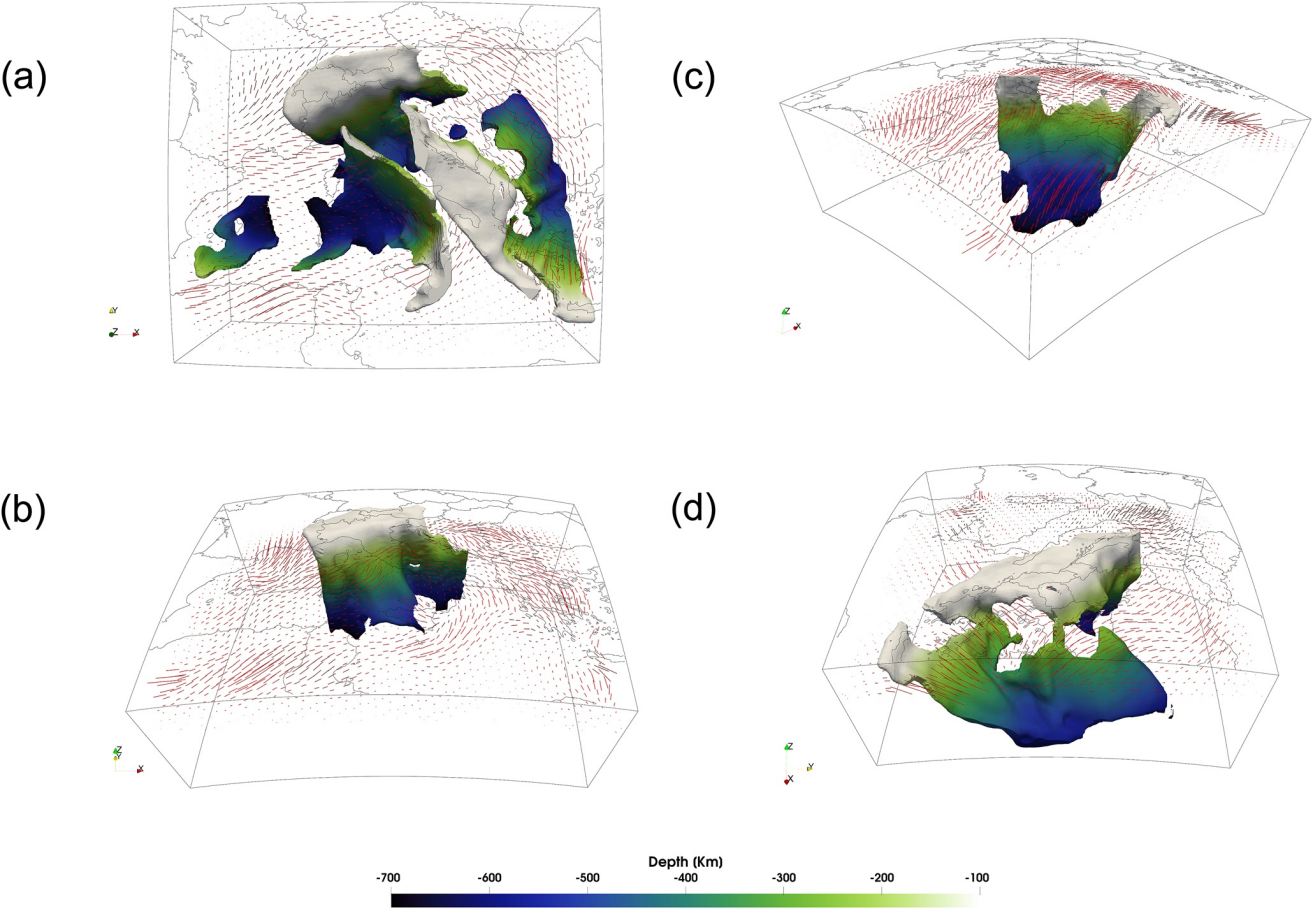
A three dimensional reconstruction of slab geometries beneath the central Mediterranean with *P*‐wave fast axes at 200 km depth. All slab segments are show from above in (a) with perspective views of the (b) Alpine, (c) Ionian, and (d) Dinaric and Hellenic slabs shown in subsequent panels. Slabs are colored by depth. Cartesian x‐, y‐, and z‐coordinates correspond to east, north, and vertical directions. Anisotropy quivers are scaled by magnitude.

Consistent with previous studies, we interpret the major fast anomalies in ani‐NEWTON21 (i.e., AlFA, ApFA, CFA, KFA, AFA, DFA, and AeFA) as descending lithospheric slabs. In Figure 6, Movie S1 and Data Set S3 in Supporting Information S1 we present a 3D model for the major slab segments present in the Central Mediterranean derived from these anomalies. Interpreting slab fragments from tomographic images can be difficult. Researchers often present either an interpretive cartoon (e.g., Hua et al., 2017; Zhao et al., 2016) or chose a single velocity contour to capture a feature of interest (e.g., Paffrath et al., 2021). The former is an idealized representation of the data, while the later can often highlight extraneous features that obscure the structures of interest. Here, we follow a modified version of the strategy outlined by Portner and Hayes (2018) to extract interpretive but data‐driven slab geometries from the tomographic model. For each potential slab fragment, the approximate trend of the slab is picked in multiple cross‐sections normal to the strike of the high‐velocity feature of interest. These slab guides are then sampled at regular 10 km intervals. At each sample point, the highest positive velocity anomaly within 100 km normal to the user‐defined slab trend is chosen as the slab core. A smooth surface is then fit to the cloud of slab core points. Finally, any tomography model node within 20 km (i.e., two node spacings) of this surface with a velocity anomaly >0.8% is considered a slab core point. Together, these slab core points produce a surface that is rendered in ParaView. In total, we identify four main slab segments (Figure 6a) which are discussed in detail below.

The AlFA is interpreted as the Alpine slab composed of European lithosphere descending southward beneath the Adriatic plate (Figures 5, 6a and 6b). The Alpine slab is further divided into an Eastern, Central, and Western segment characterized by changes in dip. The Eastern Alps slab is nearly vertical as previously imaged by several seismic tomographic models (Hua et al., 2017; Kästle et al., 2019; Koulakov et al., 2009; Lippitsch et al., 2003; Paffrath et al., 2021; Zhao et al., 2016). The ani‐NEWTON21 model does not identify any subducting body in the first 200–250 km of depth at the far eastern edge, which might indicate an ongoing shallow horizontal slab tear. The Central Alps slab dips steeply toward the southeast becoming more horizontal at ∼500 km (Figure 6b) and may be separated at depth from the eastern Alpine slab by a vertical tear (Figure 6a). However, the smoothness of the tomographic image makes it difficult to discern if the reduction in slab amplitude in this region represents a true absence of lithospheric material. The western Alpine slab dips more steeply southward relative to the central portion and does not flatten at depth. All three segments of the Alpine slab reach the transition zone. This is in contrast to previous studies which generally image a much shallower western Alpine slab (Hua et al., 2017; Kästle et al., 2019; Koulakov et al., 2009; Lyu et al., 2017; Zhao et al., 2016). However, the distinction between the northern ApFA and western AlFA become more ambiguous with depth.

At shallow depths (<∼200 km), the ApFA and DFA are interpreted as the westward and eastward descending margins of the Adria plate. The two dipping bodies reflect the double‐sided subduction of the Adria plate as described by Király et al. (2018). Deeper portions of the ApFA form a continuous band with the CFA (Figures 5a–5d) which are interpreted as the subducting and retreating Ionian lithosphere (Figures 6a and 6c). The northern most segment, anchored to the surface beneath the Northern Apennines, dives almost vertically and continuously down to a depth of ∼660 km where it begins to flatten across the mantle transition (Figure 5f) zone as predicted in models by Piromallo and Morelli (2003); Lucente et al. (2006). As described in previous studies (Amato et al., 1993; Lucente et al., 1999; Piromallo & Morelli, 2003; Selvaggi & Chiarabba, 1995; Spakman et al., 1993; Spakman & Wortel, 2004; Van der Meer et al., 2018; Zhu et al., 2012), the southern end of the Ionian slab, often referred to as the Calabrian slab, subducts north‐westward ultimately flattening and stagnating in the mantle transition zone (Figure 5g). Consistent with prior seismic imaging results (El‐Sharkawy et al., 2020; Giacomuzzi et al., 2012; Neri et al., 2009; Scarfì et al., 2018), the Calabrian slab appears to be progressively tearing apart in eastern Sicily (Figures 6a and 6c). The geometry of the window between the Northern Apennines and Calabrian slabs is widely debated. Its origin is uncertain but may have been generated from a laterally expanding tear initiated in response to the subduction of a thick continental promontory located in the Central Apennines (e.g., Lucente et al., 2006). This mechanism was reproduced numerically by Lo Bue et al. (2021) who showed that the presence of structural heterogeneities within the Adria plate may have played a role on the formation of the slab window below the Central Apennines.

The upper 200 km of the northern Dinaric slab sinks steeply toward the NE into the asthenospheric mantle (Figures 5f and 6d). At greater depths, this slab segment appears to reverse dip toward the SW. However, its true trend is difficult to discern due to the vicinity of the AlFA and a nearby fast anomaly that could also reflect the continuation of the Dinaric slab in a more NE direction. Further south, the shallow portion (<∼250 km) of the Dinaric slab continues to dive in the NE direction. We observe a laterally extensive gap from ∼200 km to ∼300 depth that separates the shallow Dinaric slab from possibly related deeper NE trending lithospheric remnants (Figures 6a and 6d). Moving toward the Aegean Sea the model captures a portion of the Hellenic slab subducting north‐eastward into the lower mantle as imaged by prior larger‐scale studies (e.g., Zhu et al., 2012). Near the surface, a gap separates the Hellenic from the Dinaric slab but they may be attached at depth (Figure 6d).

Lastly, the KFA and AFA along the northern edge of the African continent are interpreted as possible pieces of the Kabylides and Alboran slabs (Chertova et al., 2014; Van der Meer et al., 2018; van Hinsbergen et al., 2014). While these two slab fragments are separated by a large gap (Figure 6a), we note that they may be poorly resolved as they are near the less well‐instrumented southern edge of our model. Together with the ApFA, we suggest that the CFA, KFA and AFA are portions of the subducted Ionian oceanic lithosphere that since the Oligocene rolled back partly toward the italian peninsula and partly toward the Maghreb area (Chertova et al., 2014).

A variety of geodynamic processes may explain the low‐velocity zones present in our ani‐NEWTON21 model. The SFSA could reflect a thermal and or melt anomaly associated with an asthenospheric plume that may have driven Massif Central volcanic activity in the Cenozoic (Granet, Wilson, & Achauer, 1995; Granet, Stoll, et al., 1995). The upwelling could generate from buoyancy forces internal to the plume material or in response to the subduction of the Ionian plate (Faccenna et al., 2010; Yang & Faccenda, 2020). We note that the SFSA circular shape in the ani‐NEWTON21 model is more consistent with geodynamic predictions of a localized upwelling rather than the elongated SFSA shape observed in iso‐NEWTON21. The ApSA likely reflects the thermo‐chemical anomalies caused by the opening of the slab window below the Central Apennines and that led to the emplacement of the volcanic fields active in central West Italy over the last 1 My (Peccerillo, 2017). Most low velocity anomalies at shallow depths, such as the one beneath eastern Sardinia and in the Liguro‐Provençal basin, correspond to oceanic regions of the Central Western Mediterranean and can be related to the opening of back‐arc basins (Zhu et al., 2012). The AeSA is attributable to the extensional tectonic regime and continental lithosphere thinning induced by the rollback of the Hellenic slab.

Although generally the presence of low‐speed anomalies is attributed to changes in the temperature of the upper mantle, our results show how including anisotropy in the inversion greatly reduces their magnitude (Figure S11 in Supporting Information S1). Consequently, it can be deduced that not only temperature or compositional variations, but also anisotropy has a strong effect on the appearance of such anomalies in seismic images. Bezada et al. (2016) and VanderBeek and Faccenda (2021) clearly demonstrate how neglecting anisotropy could lead to errors in the interpretation of isotropic velocity models in subduction zone settings. In particular, the slow anomalies appearing below the AISA in our isotropic model iso‐NEWTON21 and in many body‐wave tomographic images and whose interpretation remained elusive, are not present in ani‐NEWTON21. The AISA is located in a region of sub‐horizontal anisotropy fabrics highlighting its potential anisotropic origin.

The location and geometry of the fast anomalies interpreted as sinking slabs do not differ substantially between the iso‐ and ani‐NEWTON21 models. An exception is the appearance of the deeper portion of the Dinaric slab in ani‐NEWTON21 as a result of the positive velocity shift occurring when the effect of the sub‐horizontal anisotropy is taken into account. At depths greater than 400 km further differences between the fast anomalies in the two models are found in the western part of the study region (Figure S11 in Supporting Information S1). As demonstrated by previous studies (e.g., Faccenna et al., 2001; Lo Bue et al., 2021) this area, at these depths, is mainly influenced by the presence of the stagnating Ionian slab lying beneath the Central Mediterranean basin as in model ani‐NEWTON21 and not beneath Southern France and Northern Spain as in model iso‐NEWTON21 where such fast anomalies appear to be artifacts due to having neglected the seismic anisotropy. The fact that fast anomalies appear to be mildly affected by including anisotropy can be explained with the strong temperature difference (up to 1000°C) likely existing between the slab cold core and the surround hot mantle. Such a thermal contrast produces strong isotropic (true) fast anomalies that are seen in both the isotropic and anisotropic inversions. On the other hand, positive thermal anomalies at sub‐lithospheric depths are on the order of few 100s °C causing relatively small variations in isotropic elastic moduli. The resulting slow velocity anomalies are thus more likely to trade‐off with anisotropic structures and, in general, different inversion methods.

### 5.2 Comparison of Anisotropic Structure With Observations

**Figure 7:**
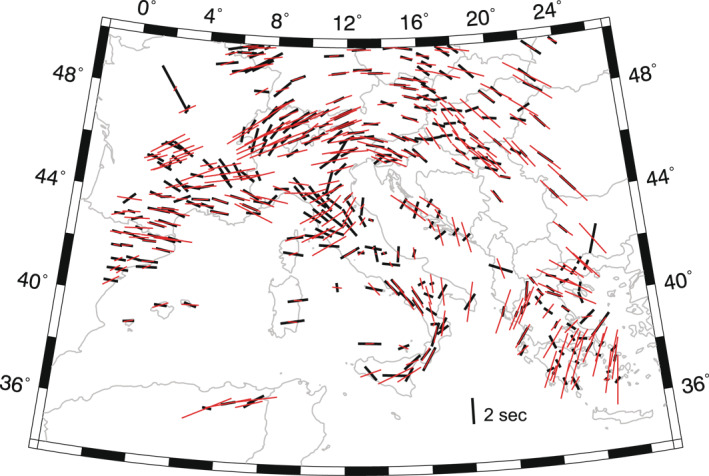
Comparison between predicted (red) and observed (black) SKS splits. Black bar at 36°N, 18°E is the 2 s scale for the splits.

Provided our ani‐NEWTON21 accurately images mantle anisotropic structure, it should be able to independently predict SKS splitting observations in the Central Mediterranean. In Figure 7, SKS splits from the database of Becker et al. (2012; updated 6 December 2020) averaged in 0.5° bins are compared to those predicted through ani‐NEWTON21 as described in Section 3.3. While SKS splits are a depth integrated measurement, they have good lateral resolution (depending on station spacing) and are primarily sensitive to upper mantle anisotropy with limited sensitivity to isotropic velocity (e.g., Sieminski et al., 2007) and thus provide an ideal basis for comparison.

While the ani‐NEWTON21 model generally predicts spatially smoother trends in fast splitting directions, they are largely consistent with the observations; the prediction error is <22° for 50% of the observations and <19° if we only consider split times ≥1 s (Figure S14 in Supporting Information S1); errors are larger by 1° if we consider station‐averaged rather than geographically binned splitting parameters. Fast directions agree well through the southern tip of the Italian peninsula into Sicily, the Alpine region and along southern margin of France into Spain. Observations and predictions also match in the Carpathians, drawing a large‐scale circular pattern that from the Bohemian Massif continues south to the Hellenic peninsula. Recent studies interpret the predominantly NW‐SE orientation also observed in the present work, as due to the compression exerted by the Adriatic plate (Qorbani et al., 2016), while the circular pattern as the result of a toroidal flow associated with the subduction of the aforementioned plate (W. Song et al., 2019).

Notable areas of disagreement include the Northern Apennines, Dinarides, and the Massif Central in southern France where errors in the predicted fast axes approach 90°. Such discrepancies could be attributed to a number of factors. For example, poorly constrained anisotropy in the upper ∼100 km. SKS splitting is biased toward the anisotropic structure at shallower depths where teleseismic *P*‐wave raypaths become more vertical and, consequently, less sensitive to anisotropy. Including local earthquake arrivals into the inversion to better constrain shallower structure could yield an anisotropic model more consistent with the SKS data. Another possibility could be changes in the mineral fabric type creating the seismic anisotropy. Here we have assumed anisotropy is largely due to olivine A‐type fabrics with a fast symmetry axis (e.g., Karato et al., 2008). However, A‐type or AG‐type fabrics (Mainprice, 2010) characterized by a slow symmetry axis are also possible which would manifest as a 90° rotation of the imaged symmetry axis in the ani‐NEWTON21 model. Elastic anisotropy from lower‐order symmetry systems (e.g., orthorhombic) could also generate discrepancies between *P*‐wave fast axes and fast SKS splitting directions. Additionally, unresolved dipping fabrics could yield orthogonal P and SKS polarization fast axes (see Figure S15 in Supporting Information S1; T.‐R. A. Song & Kawakatsu, 2012), thus explaining the discrepancies between the SKS splitting azimuths predicted from *P*‐wave models and those observed. Lastly, we acknowledge that split times are not particularly well‐fit considering the residual mean and standard deviation are −0.672 and 0.965 s, respectively (Figure S14 in Supporting Information S1). This could be related to the aforementioned factors as well as assumptions made in converting *P*‐wave anisotropy parameters into an elastic tensor (Section 3.3).

A particularly interesting area of disagreement between predicted and observed SKS fast directions is the Northern Apennines where our model predicts ENE‐WSW splitting which is nearly orthogonal to the observed NNW‐SSE trends. Our results (both in terms of SKS splitting and *P*‐wave fast azimuths) are consistent with azimuthal *P*‐wave tomography of Hua et al. (2017) who also incorporated local earthquake arrival times. Recent ambient noise tomography by Kästle et al. (2022) also constrains E‐W fast propagation directions at crustal depths suggesting the SKS splits are not likely the result of crustal fabrics. Using Pn arrivals, Díaz et al. (2013) constrain mantle anisotropic velocity structure just below the Moho and find fast *P*‐wave propagation directions that generally parallel SKS splits in the Northern Apennines. Similar orientations are also constrained by adjoint surface wave tomography in the depth range 75–125 km (Zhu & Tromp, 2013). Together, these observations suggest strong lithospheric/shallow mantle fabrics control SKS splitting and such anisotropy is not well‐constrained by our teleseismic *P*‐wave data set.

The azimuthal anisotropy component of ani‐NEWTON21 (Figure 5) is generally consistent with the larger‐scale anisotropic Rayleigh wave tomography of Zhu and Tromp (2013). For example, the sub‐circular anisotropy pattern observed by Zhu and Tromp (2013) in their model EU_60_ at 125 km of depth beneath Eastern Alps and Pannonian basin is recovered by model ani‐NEWTON21 as well and found at all depths (Figure 5). Consistently with ani‐NEWTON21 in the Central and Eastern Alps, the *P*‐wave anisotropic tomography of Hua et al. (2017) shows fast velocity directions parallel to the mountain chain from the surface down to ∼500 km of depth. The same result is confirmed by shear‐wave splitting studies of Barruol et al. (2011) and Bokelmann et al. (2013). The authors showed an arc‐shaped anisotropy pattern beneath Western and Eastern Alps, consistent with our result along the southern French coast and beneath Central and Eastern Alps. On the contrary a mismatch is observed in correspondence of the Western Alps, where Hua et al. (2017), Zhu and Tromp (2013) and Barruol et al. (2011) observe NW‐SE azimuths, interpreted by the latter as the results of the mantle flow induced by subduction and retreating and the peaks of anisotropy magnitude around the Alpine fast anomaly as induced by the sub‐lithospheric mantle deformation.

With the exception of the Southern France, model ani‐NEWTON21 (Figure 5) exhibits steep dipping fabrics mainly in the areas near subduction zones (e.g., Calabria, Alps, Northern Apennines, and Hellenic peninsula). This may be caused by the near‐vertical subduction of the slabs largely observed in the first 400 km depth, for example, in the Western Alps and Northern Apennines, that induces near‐vertical mantle flow. In agreement with the fact that positive radial anisotropy (*V*_*H*_ > *V*_*V*_) is associated with horizontal flow and negative radial anisotropy (*V*_*V*_ > *V*_*H*_) is associated with vertical flow, Hua et al. (2017) show negative *V*_*P*_ radial anisotropy (i.e., *V*_*PV*_ > *V*_*PH*_) in correspondence of dipping fast velocity anomalies, that is, close to the nearly vertical European and Adriatic slabs (i.e., Alps and Apennines). At ∼410 km depth, the model proposed by Hua et al. (2017) exhibits a widespread positive radial anisotropy (i.e., *V*_*PH*_ > *V*_*PV*_), thus a dominant horizontal mantle flow. This is confirmed by our ani‐NEWTON21 model that, despite the assumption of isotropy below 400 km depth, exhibits a gradual decrease in dip angles with respect to the shallower layers (∼<400 km) in correspondence of the main subduction zones, that may have induced nearly horizontal mantle flows.

### 5.3 Comparison of Anisotropic Structure With Geodynamic Predictions

The *P*‐wave anisotropic structure agrees well with geodynamic models of the region (e.g., Lo Bue et al., 2021), according to which the anisotropic patterns can be interpreted with sub‐vertical flows generated by subduction and the presence of horizontal flows of asthenospheric material around the main slabs related to the pressure exerted by their Mid‐to‐Late Cenozoic horizontal motion. This is true especially in the Central‐Eastern Alpine and Calabrian arcs where the fast azimuths preferentially orient trench‐parallel. In the Alpine area, this pattern can be explained with the retreat of the slabs attached to the Eurasian plate southern margin as a result of their negative buoyancy and of the compression exerted by the Adria plate. In the Calabrian arc, trench‐parallel anisotropy is likely related to the southeastward retreat of the Ionian slab (Civello & Margheriti, 2004; Faccenna et al., 2007, 2014; Jolivet et al., 2009). Similarly to what has been previously demonstrated by (Faccenda & Capitanio, 2013) on a similar synthetic scenario, the mantle below the subducting plate is subject to the pressure exerted by the slab rollback. Consequently, a horizontal flow and trench‐parallel extension establishes below the trench. The mantle flow is directed toward the slab lateral margins and beyond toward regions of lower pressure, that is, back‐arc basins, where it orients trench‐perpendicular. The predicted and observed SKS splitting data confirm the first of these two situations, while the trench‐perpendicular fabric is suggested only by our anisotropic *P*‐wave model due to the lack of receivers in the Tyrrhenian Sea for measuring SKS splitting. Therefore, the splitting measurements together with the *P*‐wave anisotropy are in agreement with what Faccenda and Capitanio (2013) observed for a similar modeled scenario. The trench‐parallel *P*‐ and SKS‐wave fast azimuths in the Dinarides can be related to (a) the NE‐oriented compression exerted by the eastern Adria plate margin causing trench‐parallel extension and seismic anisotropy, and/or the strong influence exerted by the retreating Aegean trench that is drawing mantle material from the Alpine area and toward the Aegean back‐arc basin.

## 6 Conclusions

We presented new isotropic and anisotropic tomographic images of the upper mantle in the Central Mediterranean area, obtained by inverting relative *P*‐wave delay times from teleseismic events reported by the International Seismological Centre. The primary effect of including anisotropic parameters in the inversion is a reduction in the magnitude of low‐velocity anomalies highlighting how such features could be artifacts in purely isotropic images. A three‐dimensional reconstruction of the lithospheric slabs beneath the Central Mediterranean is derived from our preferred anisotropic velocity model, ani‐NEWTON21. Three main segments of subducting lithosphere are identified belonging to the (a) Eurasian, (b) Ionian, and (c) Adria plates. These slab segments are further interrupted by a number of slab windows and tears. The entire Central Mediterranean upper mantle is characterized by substantial heterogeneity in *P*‐wave anisotropic structure particularly at 200–300 km. In general, the fast *P*‐wave azimuths trend parallel to the trenches in the foreland and progressively rotate toward the trench retreating direction in the mantle wedge/back‐arc region. These *P*‐wave derived anisotropic fabrics are largely consistent with those inferred from SKS splitting and Rayleigh wave tomography studies and with predictions from geodynamic models. Our study demonstrates the importance of including anisotropy in the inversion of teleseismic *P*‐waves as it pertains to the imaging of isotropic heterogeneity as well as generating seismic models that are consistent with other independent data sets (e.g., SKS splitting). Considering the success of this study using ISC bulletin delays, we expect higher resolution anisotropic models could be obtained using a rigorously quality controlled data set of multi‐frequency delay time measurements such as that recently generated for the Alpine region (Paffrath et al., 2021).

## Supporting information

Supporting Information S1

Movie S1

## Data Availability

Included with the publication of this manuscript are the iso‐NEWTON21 (Data Set S1 in Supporting Information S1), ani‐NEWTON21 (Data Set S2 in Supporting Information S1), and our model for central Mediterranean slabs (Data Set S3 in Supporting Information S1) all stored as NetCDF 4 files. These files are available for download via FigShare (https://figshare.com/articles/dataset/iso-_and_ani-NEWTON21_tomographic_models/19188950).
