# Supplementary material for: Slab Geometry and Upper Mantle Flow Patterns in the Central Mediterranean From 3D Anisotropic P‐Wave Tomography
Source: J Geophys Res Solid Earth. 2022 May 8;127(5):e2021JB023488. doi: 10.1029/2021JB023488 (PMC9286615; doi:10.1029/2021JB023488)
Supplement: Supplementary file 1 — Supporting Information S1 [file JGRB-127-0-s002.pdf]

[JGR – Solid Earth]

Supporting Information for

Slab geometry and upper mantle flow patterns in Central Mediterranean from 3D anisotropic P-wave tomography

F. Rappisi[1], B. P. VanderBeek[1], M. Faccenda[1], A. Morelli[2], I. Molinari[2]

[1] *Dipartimento di Geoscienze, Università di Padova, via Gradenigo 6, 35131, Padova, Italy*

[2] *Istituto Nazionale di Geofisica e Vulcanologia, via Franceschini 31, 40128, Bologna, Italy*

Contents of this file

Text S1
Text S2
Text S3
Text S4
Supplementary Figure S1
Supplementary Figure S2
Supplementary Figure S3
Supplementary Figure S4
Supplementary Figure S5
Supplementary Figure S6
Supplementary Figure S7
Supplementary Figure S8
Supplementary Figure S9
Supplementary Figure S10
Supplementary Figure S11
Supplementary Figure S12
Supplementary Figure S13
Supplementary Figure S14
Supplementary Figure S15
Supplementary Table S1

Additional Supporting Information (Files uploaded separately)

Supplementary Dataset S1
Supplementary Dataset S2
Supplementary Dataset S3
Supplementary Movie S1

Supplementary Dataset S1. iso-NEWTON21 Tomographic Model (isoNEWTON21.nc)

A NetCDF 4 file containing the iso-NEWTON21 tomographic solution with the following variables,

Longitude: A 241x201 array specifying the longitude of model points (decimal degrees)

Latitude: A 241x201 array specifying the latitude of model points (decimal degrees)

Xg: A 241x201x75 array specifying the cartesian x-coordinate of the model points (km)

Yg: A 241x201x75 array specifying the cartesian y-coordinate of the model points (km)

Zg: A 241x201x75 array specifying the cartesian z-coordinate of the model points (km)

Vpi: A 241x201x75 array specifying the starting model velocities (km/s)

dlnVp: A 241x201x75 array specifying the model relative velocity perturbations (%)

Note that the cartesian coordinates include Earth's curvature.

Supplementary Dataset S2. ani-NEWTON21 Tomographic Model (aniNEWTON21.nc)

A NetCDF 4 file containing the ani-NEWTON21 tomographic solution. The variables are the same as those described in Supplementary Dataset S1 with the following additional anisotropic variables,

Fp: A 241x201x75 array specifying the model P-wave anisotropic magnitude (%)

Psi: A 241x201x75 array specifying the model anisotropy fast-axis orientation (radians)

Gamma: A 241x201x75 array specifying the model anisotropy fast-axis elevation (radians)

Sx: A 241x201x75 array specifying the x-component of the anisotropy fast-axis vector (%)

Sy: A 241x201x75 array specifying the y-component of the anisotropy fast-axis vector (%)

Sz: A 241x201x75 array specifying the z-component of the anisotropy fast-axis vector (%)

Supplementary Dataset S3. Central Mediterranean Slab Model (Slabs_aniNEWTON21.nc)

A NetCDF 4 file containing the location of slab fragments identified in our ani-NEWTON21 tomography model. Slabs are defined on a regular 3D grid where grid node values of 1 indicate that point is located within the core of the slab and values of 0 indicate the point is outside the slab core. See Section 5.1 of Rappisi et al. (2022) for description of slab identification,

Longitude: A 241x201 array specifying the longitude of model points (decimal degrees)

Latitude: A 241x201 array specifying the latitude of model points (decimal degrees)

Xg: A 241x201x75 array specifying the cartesian x-coordinate of the model points (km)

Yg: A 241x201x75 array specifying the cartesian y-coordinate of the model points (km)

Zg: A 241x201x75 array specifying the cartesian z-coordinate of the model points (km)

ApennineCalabria: A 241x201x75 array identifying points as inside (1) or outside (0) the Apennine and Calabrian slabs

Alpine: A 241x201x75 array identifying points as inside (1) or outside (0) the Alpine slab

DinaricHellenic: A 241x201x75 array identifying points as inside (1) or outside (0) the Dinaric and Hellenic slabs

African: A 241x201x75 array identifying points as inside (1) or outside (0) slab fragments located along the African margin

Note that the cartesian coordinates include Earth's curvature.

Supplementary Movie S1. A 3D reconstruction of slab geometries beneath the central Mediterranean with P-wave fast axes shown by quivers (scaled by anisotropic magnitude) at 200 km depth.

Text S1. Selection of regularization parameters

To identify appropriate regularization values (i.e. damping and smoothing factors, λ_d and λ_s, respectively), we constructed L-curves (Figure S2) which plot the squared-norm of the data residual vector against the squared-norm of the model perturbational vector.

The model norm comprises the fractional velocity perturbations (i.e. the change in velocity with respect to the starting model inversely weighted by the initial model velocities) and the anisotropic magnitude perturbations both of which are on the order of a few percent. Because both values describe changes in velocity, they are directly comparable.

Ideal solutions are considered those near the corner of the L-curve where an increase in model norm does not result in an appreciable decrease in data residuals.

We first ran a series of purely isotropic inversions at different λ_d values for different fixed ratios of λ_s/λ_d such that the relative influence of damping versus smoothing on each solution remained constant. The preferred λ_s/λ_d ratio was selected such that further reductions in this ratio did not yield appreciable reductions in data residuals while smoother solutions more rapidly degraded the data fit and generally required a greater number of perturbations to achieve similar RMS residual values relative to less smooth solutions (Figure S2). A series of anisotropic inversions were subsequently run in which the damping applied to the mean P-wave slowness perturbations was varied across different ratios of slowness-to-anisotropic parameter damping assuming the same λ_s/λ_d for both parameter sets identified from the aforementioned isotropic inversions. Considering that both isotropic and anisotropic heterogeneity are likely significant beneath the study area, we chose the anisotropic damping ratio such that the norm of the fractional velocity perturbations equals the norm of the anisotropic magnitude perturbations. The L-curve for the anisotropic inversions using this ratio is shown in Figure S2. The parameters adopted for our preferred isotropic and anisotropic solutions are shown in Table S1.

Text S2. Model resolution

Anisotropic imaging with teleseismic delay times has some important limitations. Because the delay times are demeaned, they only illuminate changes in velocity relative to an unknown average value. The implication for anisotropic imaging is that there may be a systematic bias in the recovered anisotropic parameters as demeaning effectively removes the average back-azimuthal signal in delay times. For this reason, a laterally homogeneous isotropic or anisotropic layer spanning the entire model cannot be imaged. However, provided that the imaging volume contains heterogeneous anisotropic structure, this back-azimuth trend will amount to a constant value and not impart any bias on the recovered anisotropic parameters. See Section 4 of VanderBeek and Faccenda (2021) for further discussion. Considering the variety of splitting parameters observed across the central Mediterranean, the mantle appears sufficiently heterogeneous such that anisotropy can be accurately recovered--an inference that has been verified through our synthetic tests.

The steep incidence angles of teleseismic ray paths also adversely affect model resolution. In particular, resolution is poor at depths ~ 75 km where rays are steepest and ray crossing is limited. Therefore, anomalies in the uppermost lithosphere are not interpreted. Limited sampling of incidence angles can result in poor vertical resolution of anisotropic fabrics. However, good azimuthal teleseismic ray coverage is sufficient to constrain anisotropic domains characterized by horizontal or dipping fabrics (VanderBeek and Faccenda, 2021). Another consequence of restricted incidence angle coverage is that the full magnitude of directional velocity variations are not sampled causing an underestimation of anisotropy strength as shown in our synthetic tests. Lastly, with imperfect data coverage, trade-offs between isotropic and anisotropic parameters are likely. However, the trade-off appears to be largely one-sided in that unaccounted for or poorly imaged anisotropic heterogeneity creates significant isotropic artefacts but truly isotropic structure does not tend to generate anisotropic artefacts as demonstrated by VanderBeek and Faccenda (2021) and our own synthetic tests.

The derivative weight sum (DWS; Toomey & Foulger 1989), i.e. the summation of travel-time partial derivatives with respect to slowness at each perturbational node, provides an indirect estimate of parameter resolution attaining higher values in more densely sampled regions of the model. Based on the checkerboard resolution tests discussed in Section 3.3 of the main text, we find that isotropic structure in areas with DWS > 100 is generally well-recovered. We present maps of the DWS in Figure S3 from which we can see that the upper mantle beneath Italy and mainland Europe is well sampled by teleseismic rays. In contrast, the dearth of stations throughout the Mediterranean Sea and north Africa results in a clear reduction in DWS and by inference less well-resolved velocity structure. However, teleseismic rays travelling to stations throughout mainland Europe do illuminate this area at depths greater than ~150 km.

The DWS lacks information regarding how directionally well-sampled are the model parameters which is important for assessing resolution of anisotropic structure. To assess directional bias, we use the azimuthal mean resultant length (AMRL; Fisher, 1995; Zhang et al., 2009) defined as the length of the vector resulting from an averaging of the x- and y-components of all ray segment unit vectors sampling a given perturbational node. If a node is directionally well-sampled, the AMRL tends toward zero while the AMRL will approach 1 in areas with a strong directional bias. Based on our checkerboard tests (Section 3.3), AMRL < 0.5 indicates a node is sufficiently directionally sampled to image anisotropic structure. Maps of the AMRL for our study are shown in Figure S4. It is found that the AMRL reflects the DWS (Figure S3) indicating that areas of high data density correspond to areas of good directional coverage. The Mediterranean Sea region is primarily sampled by rays coming from the south and east, and travelling to stations on mainland Europe resulting in high AMRL values.

Text S3. Resolution of dipping fabrics

To further investigate the resolution of dipping fabrics, we performed two additional synthetic tests for three 300 km x 300 km x 200 km anisotropic anomalies centered at 200 km depth beneath the Western Alps, Dinarides, and Calabria--three areas with unique anisotropic fabrics in the preferred tomographic solution. We prescribed 6% anisotropy to these three anomalies with an azimuth at a high-angle to that imaged in the preferred model and created two synthetic datasets for shallowly (30°) and steeply (60°) dipping fabrics. The true and recovered anomalies are shown in Figure S6 and S7. Both the azimuth and dips are well-imaged for both synthetic models with amplitudes being more under-estimated for the steeper fabrics.

Text S4. SKS from P-wave tomography

We use the method of Rümpker and Silver (1998) to model the effect of anisotropy on a SKS waveform approximated as a Ricker wavelet with a central period of 10 s. The method involves progressively rotating and time shifting a waveform, initially linearly polarised in the back-azimuth direction, through a series of anisotropic layers. The splitting parameters (i.e. time delay and fast direction) are computed on the resulting waveform following Silver and Chan (1991). To compute the time shifts for each layer, we use the Christoffel equations and take into account variations in ray incidence. The elastic tensor in principle coordinates at every point in our model is defined from the P-wave velocities as:

$$
\begin{bmatrix}
[v_p(1-f_p)]^2 & C_{11}-2C_{66} & C_{11}-2C_{66} & 0 & 0 & 0 \\
 & [v_p(1-f_p)]^2 & C_{11}-2C_{66} & 0 & 0 & 0 \\
 & & [v_p(1+f_p)]^2 & 0 & 0 & 0 \\
 & & & [gv_p(1+hf_p)]^2 & 0 & 0 \\
 & \text{Symmetry} & & & [gv_p(1+hf_p)]^2 & 0 \\
 & & & & & [gv_p(1-hf_p)]^2
\end{bmatrix}
$$

where v_p is the mean isotropic P-wave velocity; f_p is the anisotropic magnitude; g is a constant compressional-to-shear wave speed ratio assumed here to be 1.81; and h is a constant compressional-to-shear wave anisotropy magnitude ratio assumed to be 1.51. These values were taken from laboratory measurements of a peridotite sample by Kern (1993) who found $C_{12} \approx C_{13}$ and $C_{13} = C_{23}$. The tensor is then rotated such that the C_{33}-direction parallels the P-wave fast direction in our model. SKS splits are computed for all stations in the study area with observations listed in the compilation of Becker et al. (2012 updated 2020). Ray paths are predicted via the TauP toolkit Crotwell et al. (1999) using the reported event-station back-azimuths and ranges. Ray paths are discretised into 10 km segments through which the splitting effect is computed. We present the results of this analysis in the discussion of our preferred tomographic models (Section 5.1) and simply note here that our P-wave model predicts many patterns observed in prior SKS studies.

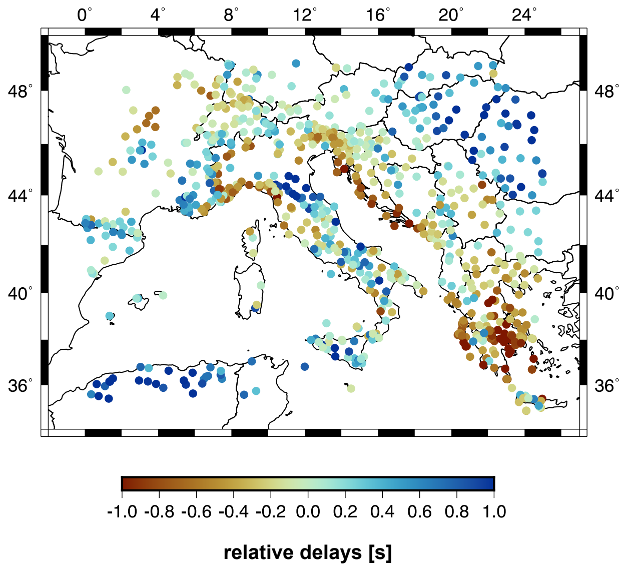

Supplementary Figure S1. Mean Station delays with respect to model AK135.

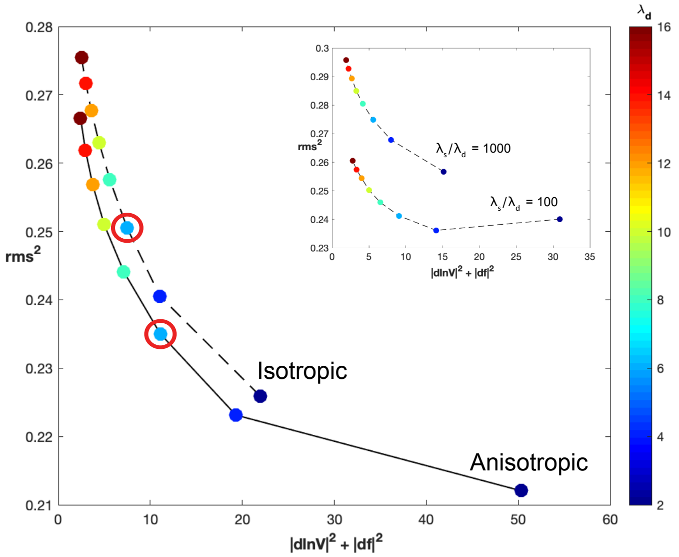

Supplementary Figure S2. Trade-off curves for damping and smoothing multiplier selection. The squared RMS is plotted as function of the squared model norm, $|dlnV|^2 + |df|^2$, where dlnV is the fractional velocity perturbation vector and df is the anisotropic magnitude perturbation vector. The dashed and solid lines are, respectively, the trade-off curve for purely isotropic and anisotropic inversion. The values λ_s/λ_d are kept fixed at 200 for both the isotropic and anisotropic cases. Colorbar represents different values of damping factor for slowness. Red circles indicate the preferred solutions presented in section 4, corresponding to $\lambda_d = 6$. Insert shows the trade-off curves from two isotropic tests with $\lambda_s/\lambda_d = 100$ and 1000.

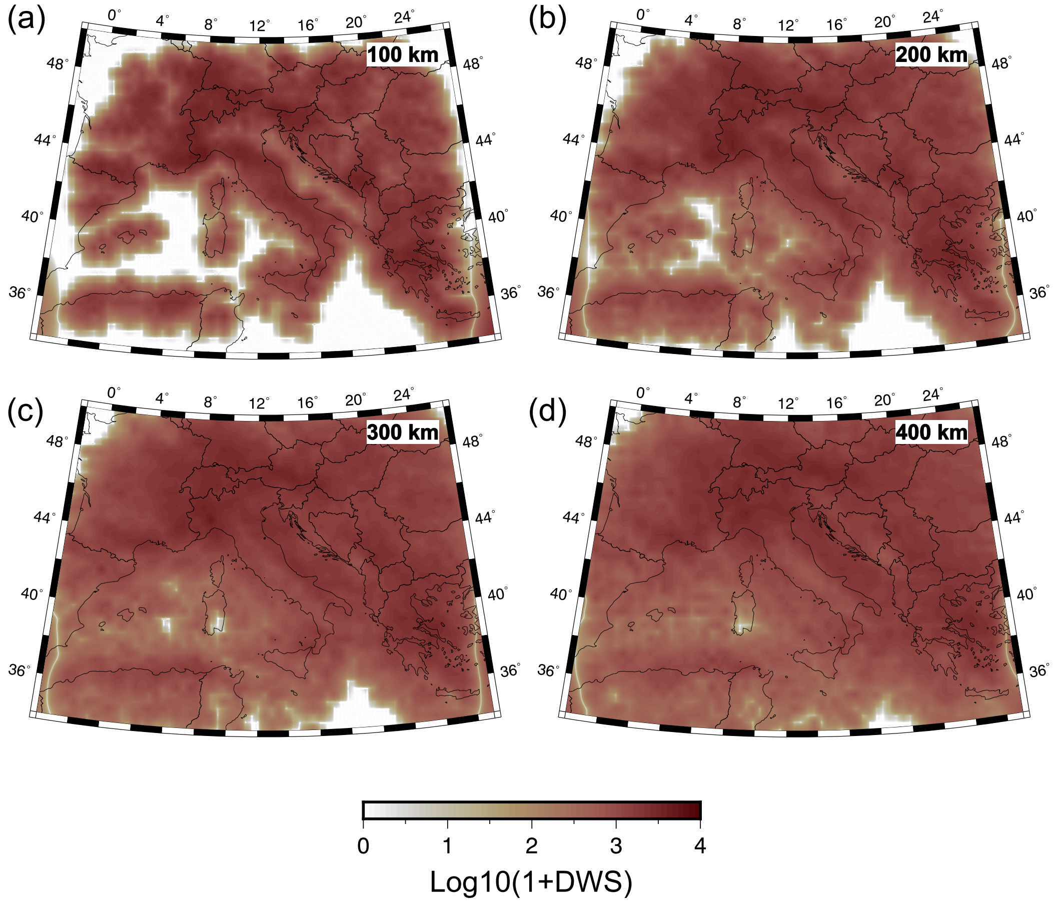

Supplementary Figure S3. Derivative weighted sum (DWS) at (a) 100 km, (b) 200 km, (c) 300 km, (d) 400 km depth.

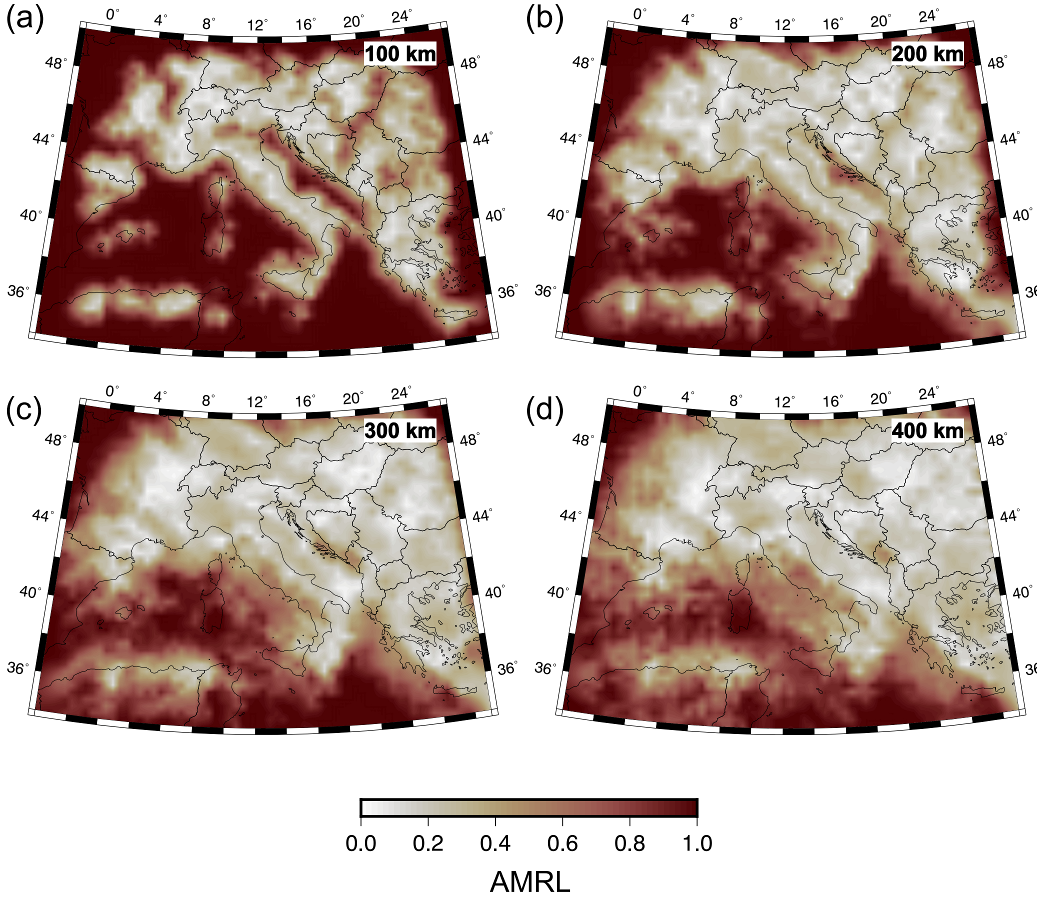

Supplementary Figure S4. Azimuthal mean resultant length (AMRL) at (a) 100 km, (b) 200 km, (c) 300 km, (d) 400 km depth.

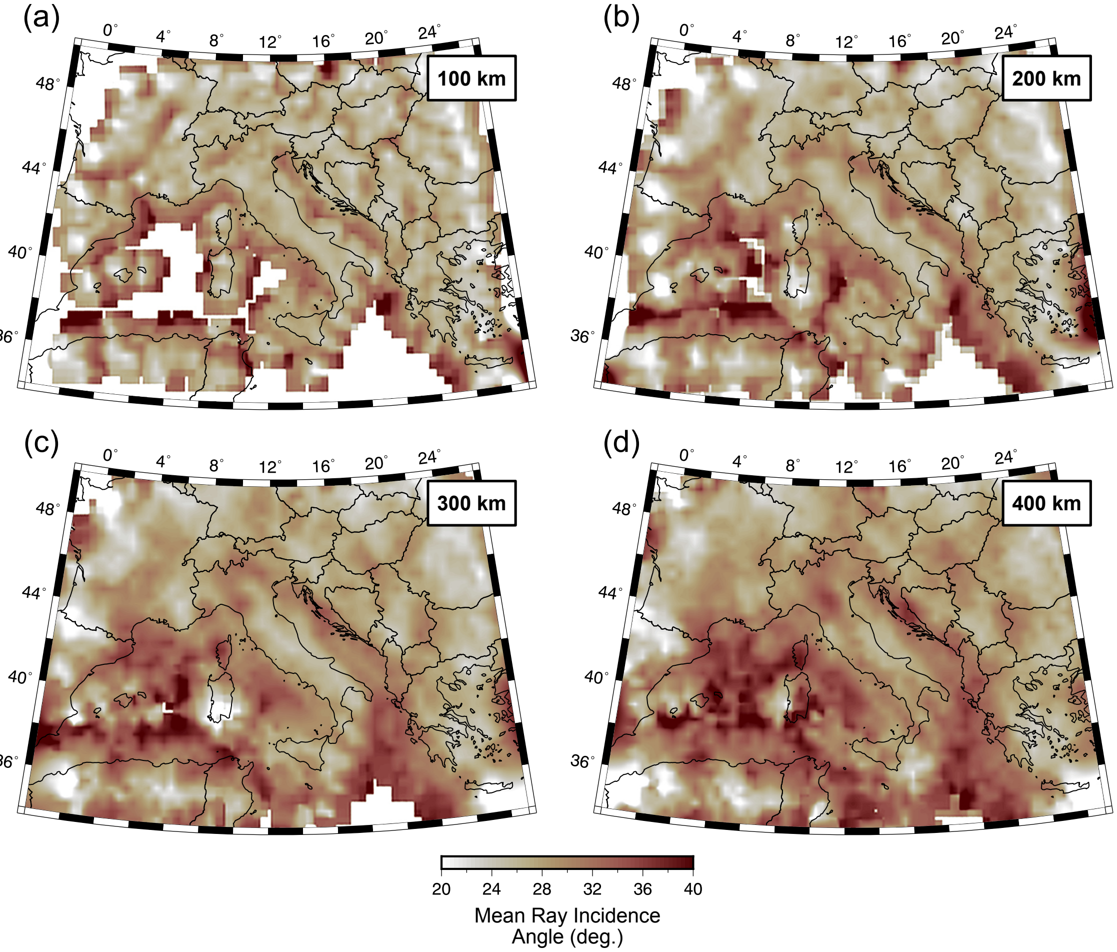

Supplementary Figure S5. Mean ray incidence angles at (a) 100 km, (b) 200 km, (c) 300 km, and (d) 400 km depth.

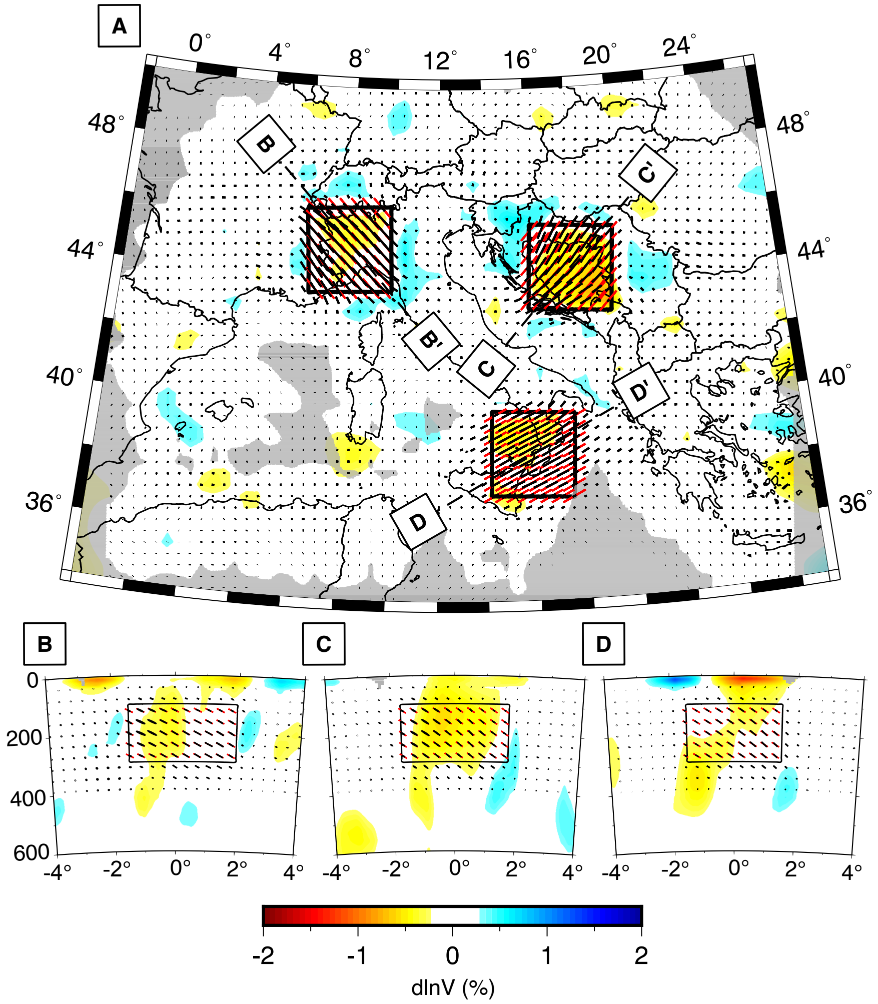

Supplementary Figure S6. Recovery of shallow dipping (30°) anisotropic fabrics. The true anisotropic anomalies have dimensions of 300 km x 300 km x 200 km and are centered at 200 km depth; no isotropic heterogeneity is present in the true model. (A) Map view at 200 km depth of imaged anisotropic and isotropic structure. Cross-section through the (B) Western Alps, (C) Dinarides, and (D) Calabrian anomalies are plotted along the corresponding cross-section lines in (A). In all panels, red quivers show the true anisotropic fabric orientations whose length is scaled by the anisotropic magnitude (6%) and the black box outlines the extent of the anomalies. Black quivers are the recovered anisotropic orientations.

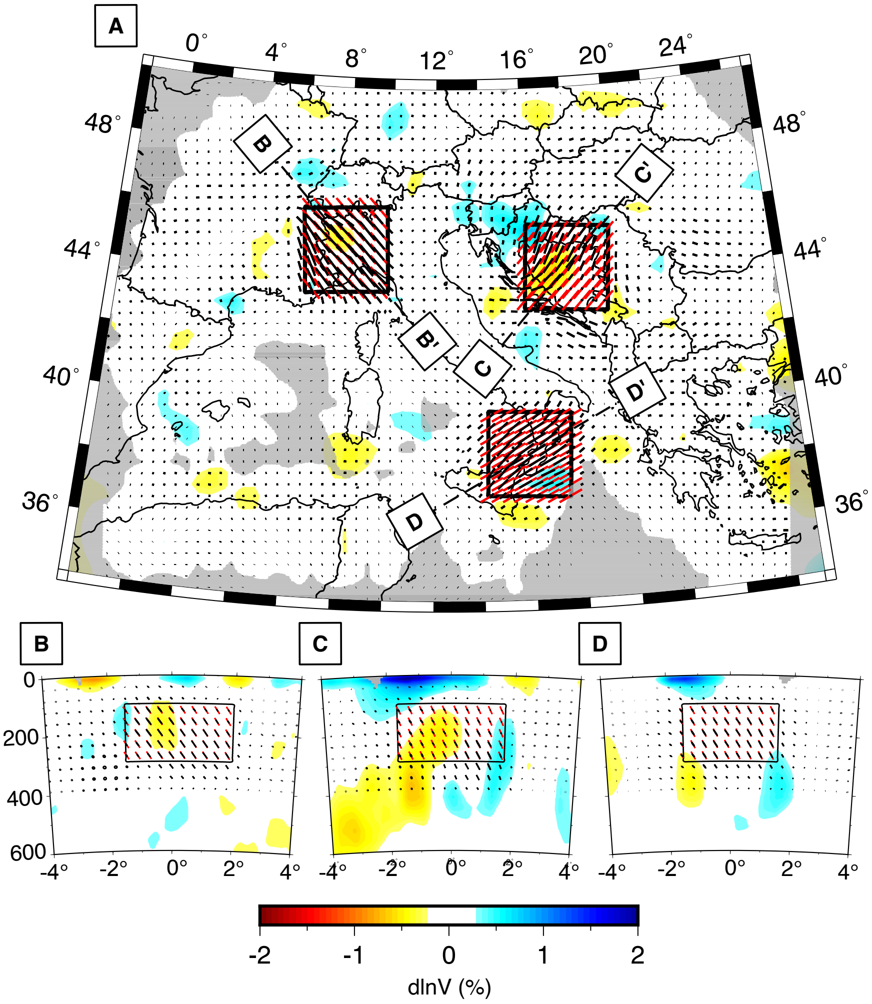

Supplementary Figure S7. Same as Figure S6 but for steeply dipping (60°) anisotropic fabrics.

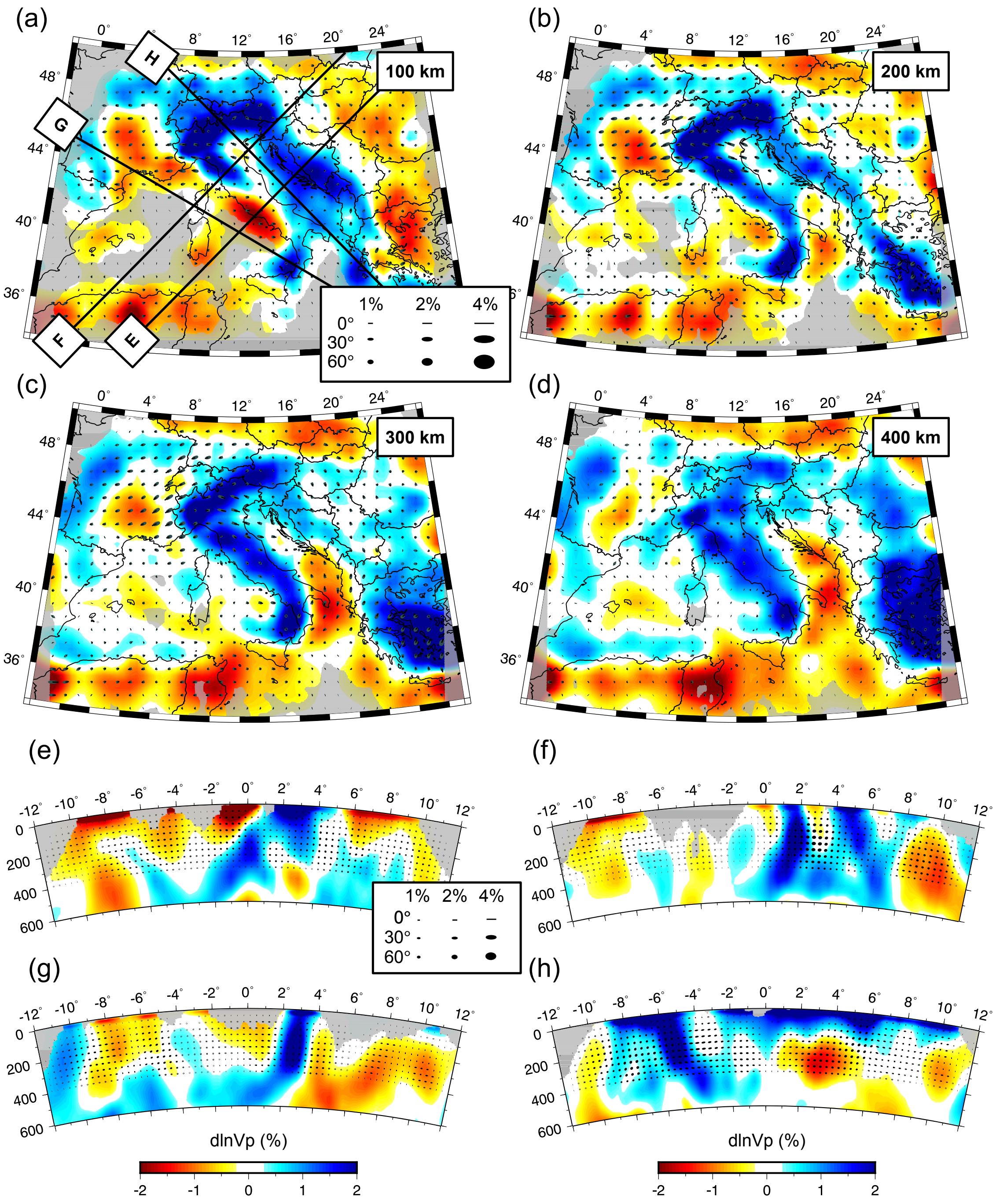

Supplementary Figure S8. Isotropic restoration synthetic test (see Section 3.3 for details). Anisotropic inversion for our preferred isotropic model depicted in Figure 4. While no anisotropic structure is present in the target model, the inversion does introduce some anisotropic perturbations. Anisotropy is represented by ellipse symbols where the major axis of the ellipse parallels the fast-direction and the minor axis scales linearly with the symmetry axis dip into the view plane such that fabrics parallel and normal to the cross-sections plot as lines and circles, respectively.

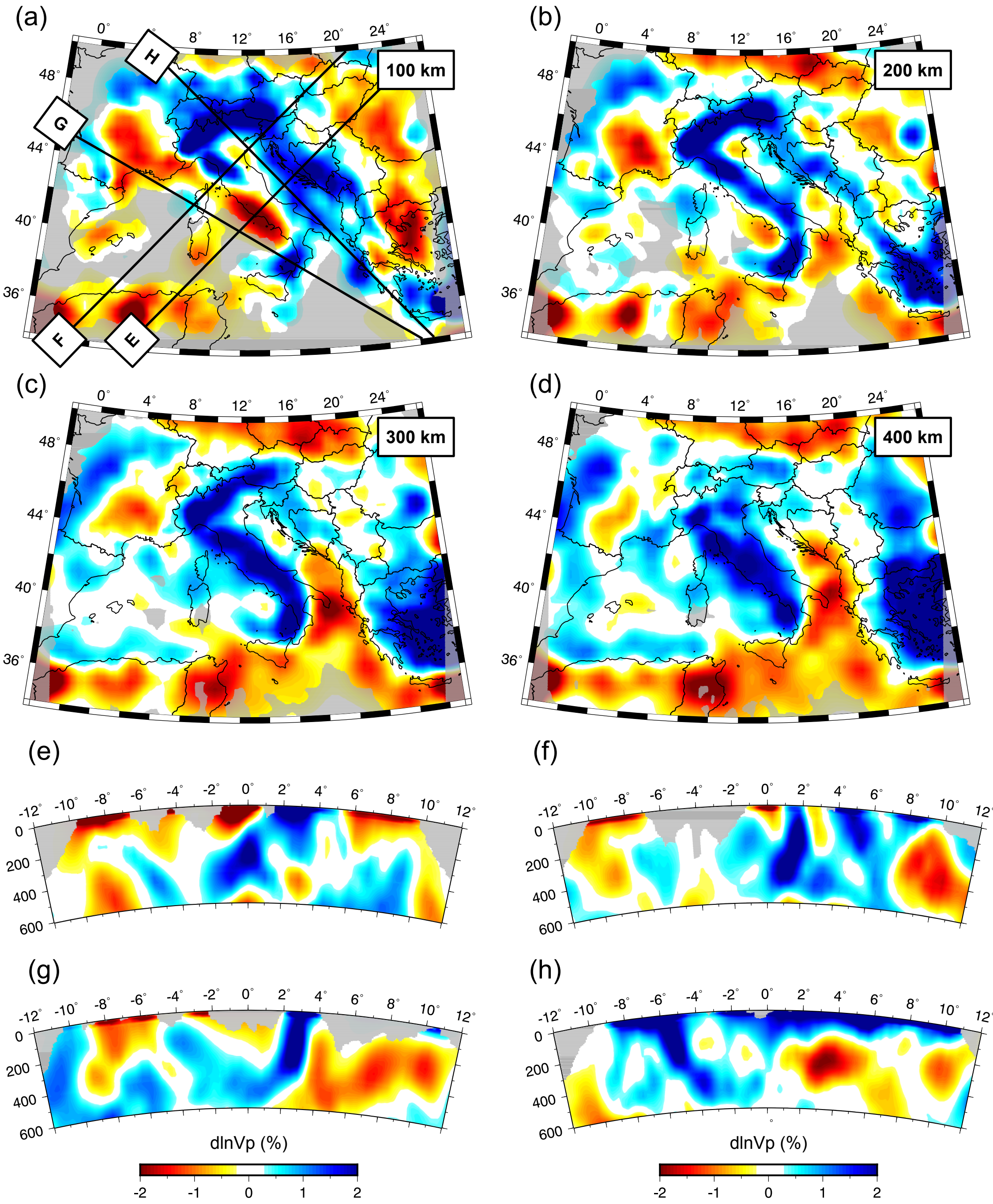

Supplementary Figure S9. Anisotropic restoration synthetic test (see Section 3.3 for details). Isotropic inversion for our preferred anisotropic model depicted in Figure 5. Note similarity of this solution to the preferred isotropic model (Figure 4).

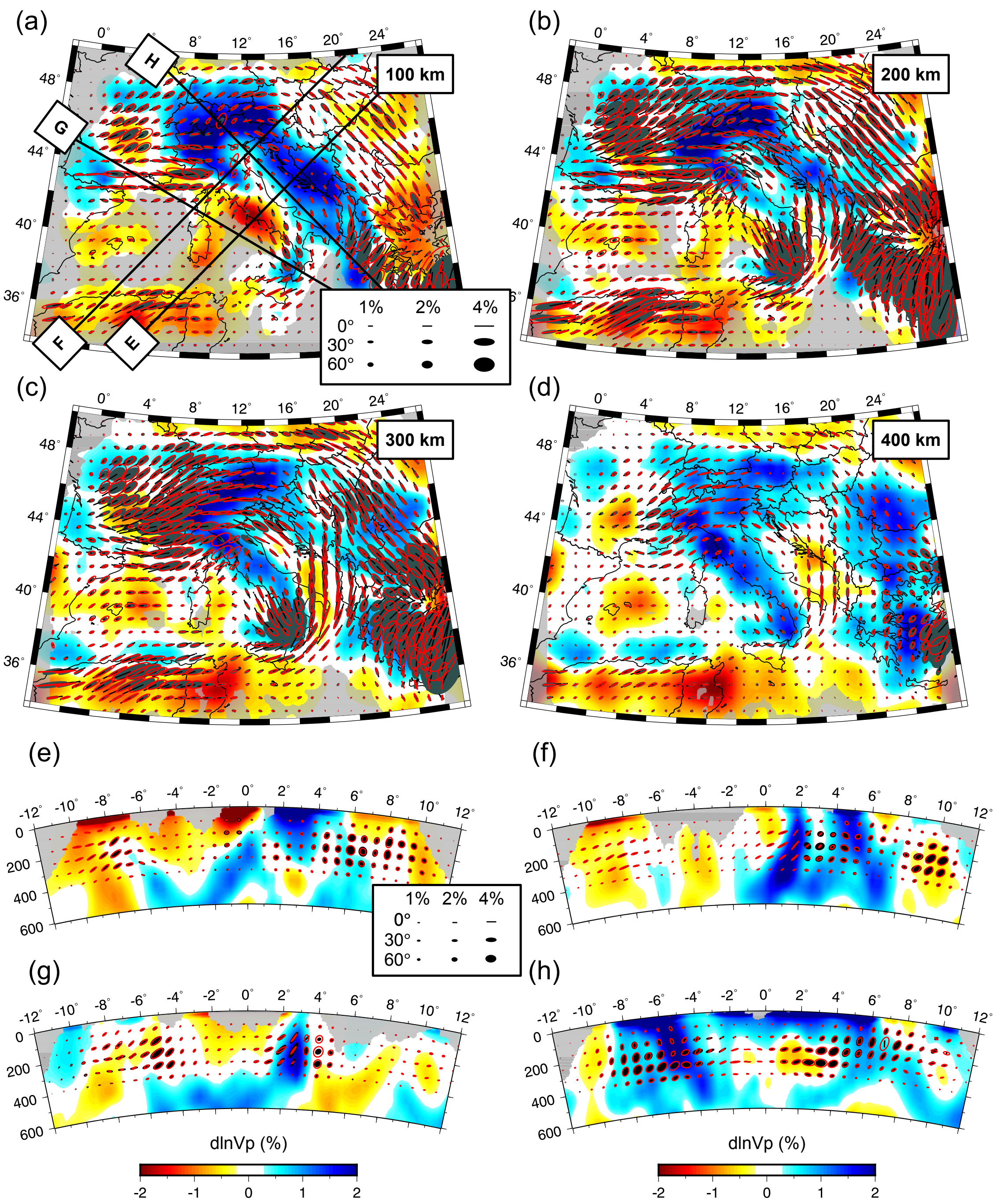

Supplementary Figure S10. Anisotropic restoration synthetic test (see Section 3.3 for details). Anisotropic inversion for our preferred anisotropic model depicted in Figure 5. Anisotropy is represented by ellipse symbols where the major axis of the ellipse parallels the fast-direction and

the minor axis scales linearly with the symmetry axis dip into the view plane such that fabrics parallel and normal to the cross-sections plot as lines and circles, respectively. For ease of comparison, the true/target anisotropic fabrics are shown by the red ellipses.

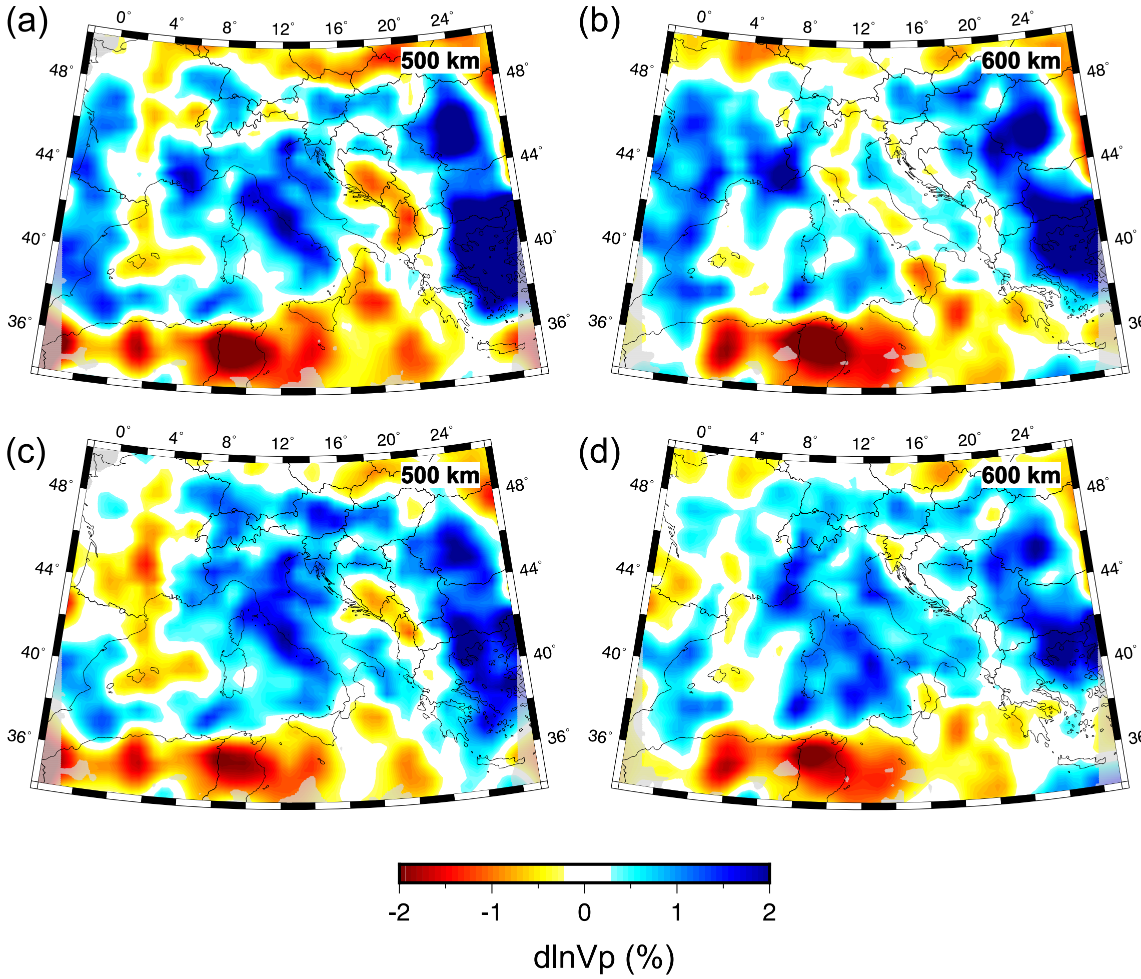

Supplementary Figure S11. Horizontal map views at 500 km and 600 km depth for model iso-NEWTON21 (a, b) and ani-NEWTON21 (c, d). P-wave velocity anomalies shown with respect to reference model AK135.

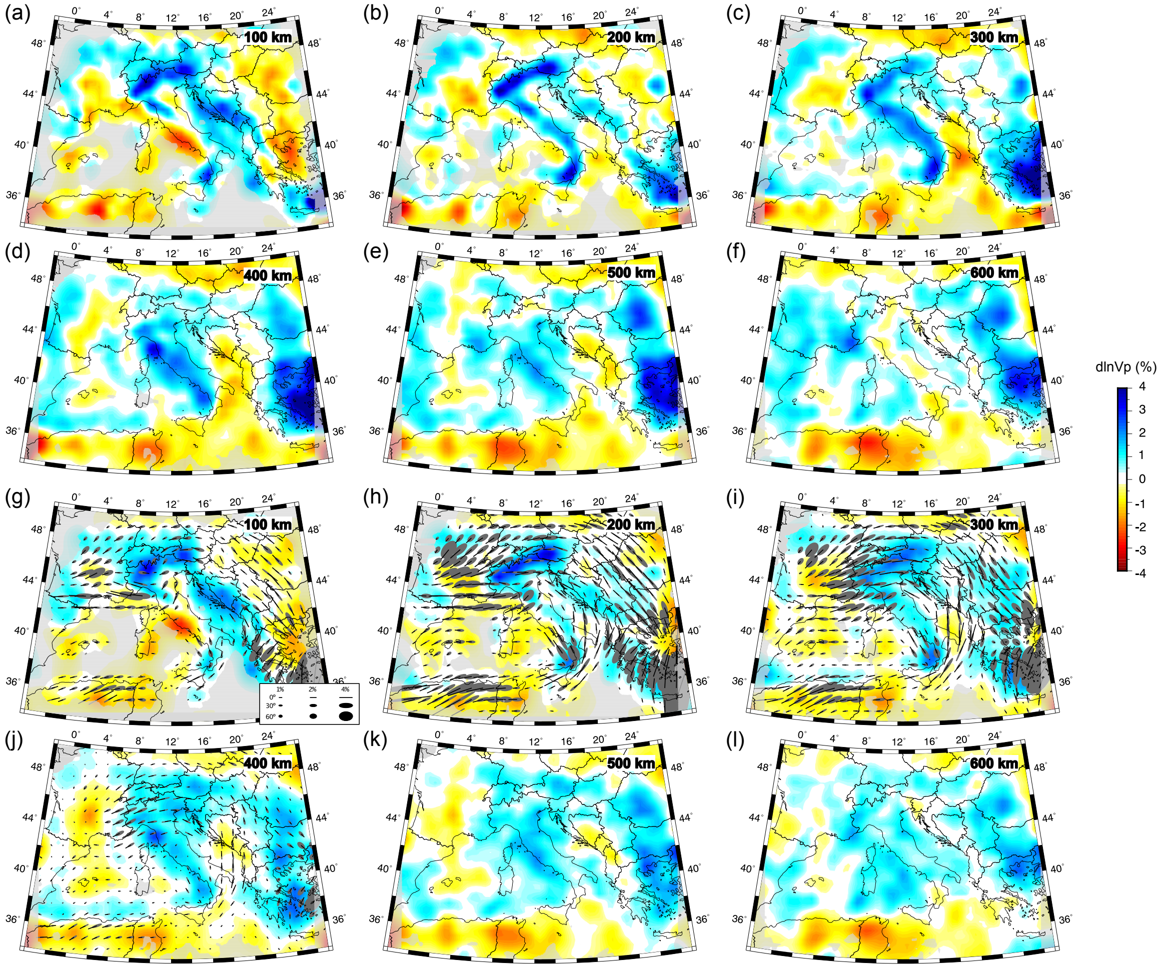

Supplementary Figure S12. (a-f) Iso-NEWTON21 and (g-l) ani-NEWTON21 with broader colorscale limits with respect to Figure 4 and Figure 5. Horizontal cross-sections are shown at (a,g) 100 km, (b,h) 200 km, (c,i) 300 km, (d,j) 400 km, (e,k) 500 km and (f,l) 600 km depth. Isotropic anomalies are plotted with respect to starting model. Anisotropy is represented by ellipse symbols where the major axis of the ellipse parallels the fast-direction and the minor axis scales linearly with the symmetry axis dip into the view plane such that fabrics parallel and normal to the cross-sections plot as lines and circles, respectively. Areas of poor data coverage are masked in grey.

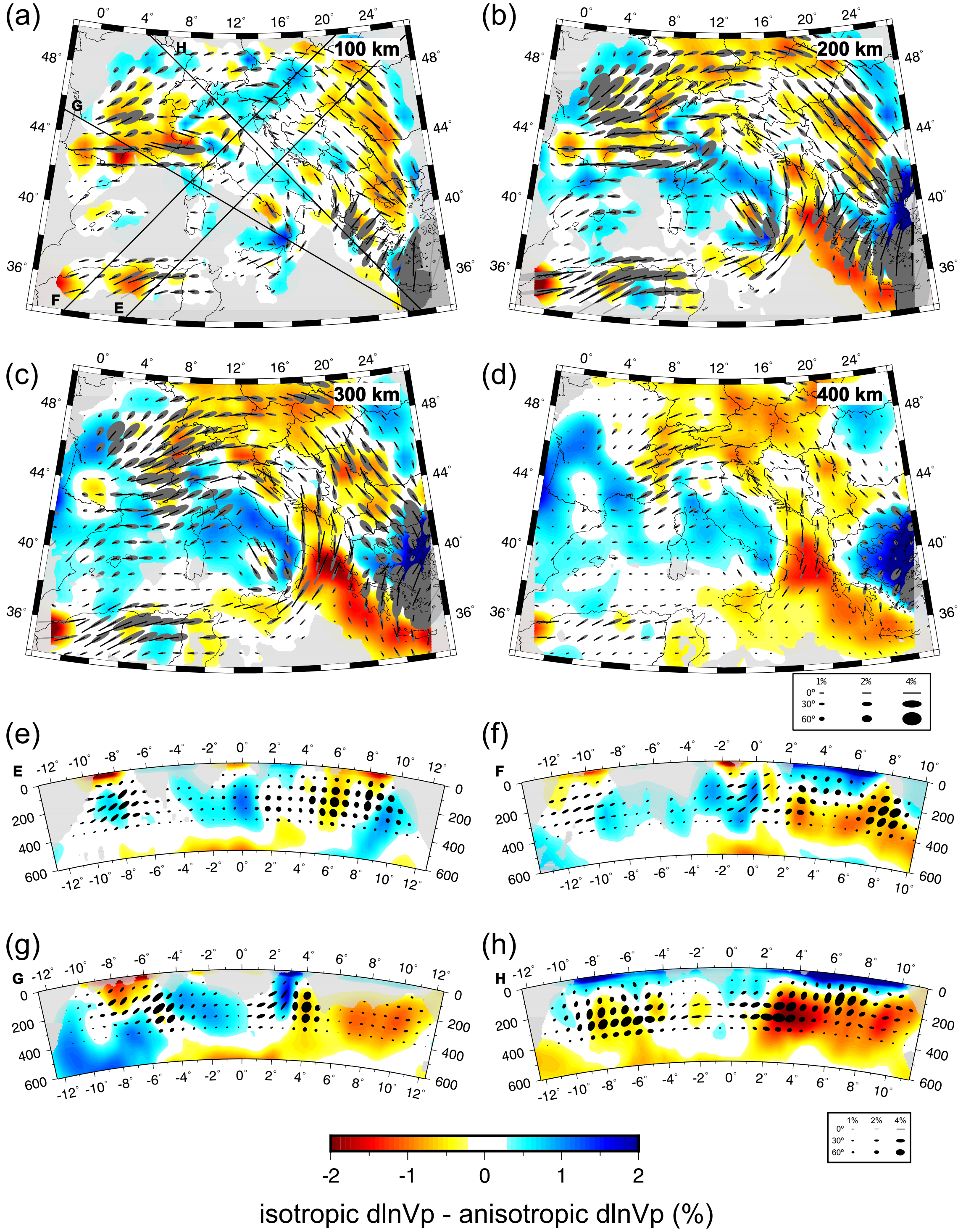

Supplementary Figure S13. Differences between iso-NEWTON21 and ani-NEWTON21 isotropic anomalies. Horizontal cross-sections are shown at (a) 100 km, (b) 200 km, (c) 300 km, and (d) 400 km depth. Vertical cross-sections are shown in (e-f) along the corresponding profile lines drawn in (a). Anisotropy from ani-NEWTON21 is represented by ellipse symbols where the major axis of the ellipse parallels the fast-direction and the minor axis scales linearly with the symmetry axis dip into

the view plane such that fabrics parallel and normal to the cross-sections plot as lines and circles, respectively. Areas of poor data coverage are masked in grey.

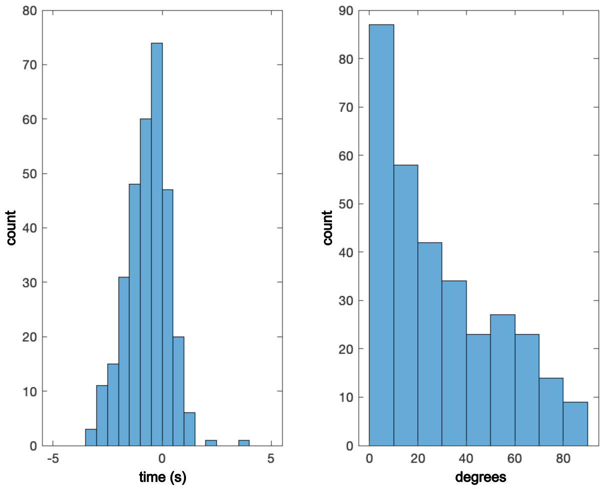

Supplementary Figure S14. Time (left) and azimuth (right) errors between predicted and observed SKS splitting measurements. The split time mean error is -672 ms with a standard deviation of 965 ms; the split azimuth median error is 22 for the entire dataset 19 if only considering split times greater than 1 s.

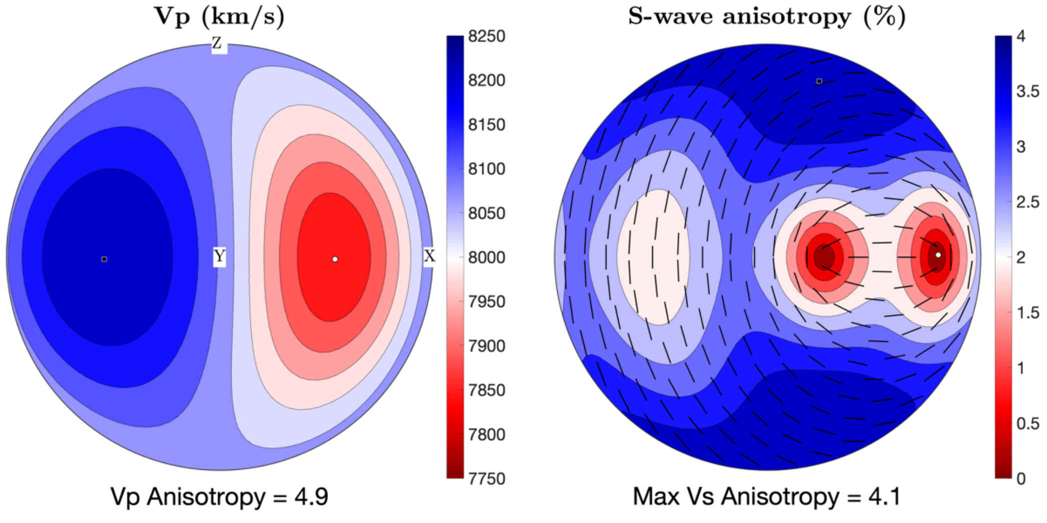

Supplementary Figure S15. Seismic velocities pole projections for an elastic tensor representative of the asthenospheric mantle as defined in Song and Kawakatsu (2012). The tensor is characterized by orthorhombic symmetry and has been rotated by 45° around the N-S axis. (left) P-wave velocity in m/s; (right) S wave anisotropy (%) = $(Vs_1 - Vs_2)/(Vs_1 + Vs_2) * 200$. The black bars indicate the azimuth of the fast S-wave component for different seismic ray propagation directions. In both panels, the black square and white circle indicate, respectively, the seismic ray direction yielding max. and min. values. For teleseismic rays with high incidence angles (i.e., close to the center of

the circle) the azimuth of the fast S-wave is N-S, which is at 90° from the fast P-wave oriented in the E-W plane. This discrepancy is found for dipping angles > 30°. Pole projections plotted with MTEX (Mainprice et al., 2011).

	λ_s/λ_d	λ_d	λ_s	data	rms	dm norm	iterations
iso-NEWTON21	200	6	1200	isotropic	0.5	2.73	3
ani-NEWTON21	200	6	1200	anisotropic	0.48	4.64	6

Supplementary Table S1. Inversion parameters summary table. Damping (λ_d) and smoothing (λ_s) factors, type of data (isotropic/anisotropic), rms, perturbation vector (dm) norm (calculated as |dlnV| + |df|, where dlnV is the fractional velocity perturbation vector and df is the anisotropic magnitude perturbation vector), iteration at convergence.

Reference

Becker, T. W., Lebedev, S., & Long, M. D. (2012). On the relationship between azimuthal anisotropy from shear wave splitting and surface wave tomography. Journal of Geophysical Research: Solid Earth, 117(B1).

Crotwell, H. P., Owens, T. J., & Ritsema, J. (1999). The TauP Toolkit: Flexible seismic travel-time and ray-path utilities. Seismological Research Letters, 70, 154-160.

Fisher, N. I. (1995). Statistical analysis of circular data. cambridge university press.

Kern, H. (1993). P-and S-wave anisotropy and shear-wave splitting at pressure and temperature in possible mantle rocks and their relation to the rock fabric. Physics of the Earth and Planetary Interiors, 78(3-4), 245-256.

Mainprice, D., Hielscher, R., & Schaeben, H. (2011). Calculating anisotropic physical properties from texture data using the MTEX open-source package. Geological Society, London, Special Publications, 360(1), 175-192.

Rümpker, G., & Silver, P. G. (1998). Apparent shear-wave splitting parameters in the presence of vertically varying anisotropy. Geophysical Journal International, 135(3), 790-800.

Song, T. R. A., & Kawakatsu, H. (2012). Subduction of oceanic asthenosphere: Evidence from sub-slab seismic anisotropy. Geophysical Research Letters, 39(17).

Silver, P. G., & Chan, W. W. (1991). Shear wave splitting and subcontinental mantle deformation. Journal of Geophysical Research: Solid Earth, 96(B10), 16429-16454.

Toomey, D. R., & Foulger, G. R. (1989). Tomographic inversion of local earthquake data from the Hengill-Grensdalur central volcano complex, Iceland. Journal of Geophysical Research: Solid Earth, 94(B12), 17497-17510.

VanderBeek, B. P., & Faccenda, M. (2021). Imaging upper mantle anisotropy with teleseismic P-wave delays: insights from tomographic reconstructions of subduction simulations. Geophysical Journal International, 225(3), 2097-2119.

Zhang, H., Thurber, C., & Bedrosian, P. (2009). Joint inversion for vp, vs, and vp/vs at SAFOD, Parkfield, California. Geochemistry, Geophysics, Geosystems, 10(11).
